# Antigiardial activity of novel triazolyl-quinolone-based chalcone derivatives: when oxygen makes the difference

**DOI:** 10.3389/fmicb.2015.00256

**Published:** 2015-04-08

**Authors:** Vijay Bahadur, Daniela Mastronicola, Amit K. Singh, Hemandra K. Tiwari, Leopoldo P. Pucillo, Paolo Sarti, Brajendra K. Singh, Alessandro Giuffrè

**Affiliations:** ^1^Bio-Organic Laboratory, Department of Chemistry, University of DelhiDelhi, India; ^2^CNR Institute of Molecular Biology and PathologyRome, Italy; ^3^Department of Biochemical Sciences and Istituto Pasteur – Fondazione Cenci Bolognetti, Sapienza University of RomeRome, Italy; ^4^L. Spallanzani National Institute for Infectious Diseases, Istituto di Ricovero e Cura a Carattere ScientificoRome, Italy

**Keywords:** chemical synthesis, drug screening, anaerobic protozoa, intestinal disease, microaerobiosis

## Abstract

Giardiasis is a common diarrheal disease worldwide caused by the protozoan parasite *Giardia intestinalis*. It is urgent to develop novel drugs to treat giardiasis, due to increasing clinical resistance to the gold standard drug metronidazole (MTZ). New potential antiparasitic compounds are usually tested for their killing efficacy against *G. intestinalis* under anaerobic conditions, in which MTZ is maximally effective. On the other hand, though commonly regarded as an ‘anaerobic pathogen,’ *G. intestinalis* is exposed to relatively high O_2_ levels *in vivo*, living attached to the mucosa of the proximal small intestine. It is thus important to test the effect of O_2_ when searching for novel potential antigiardial agents, as outlined in a previous study [Bahadur et al. (2014) Antimicrob. Agents Chemother. 58, 543]. Here, 45 novel chalcone derivatives with triazolyl-quinolone scaffold were synthesized, purified, and characterized by high resolution mass spectrometry, ^1^H and ^13^C nuclear magnetic resonance and infrared spectroscopy. Efficacy of the compounds against *G. intestinalis* trophozoites was tested under both anaerobic and microaerobic conditions, and selectivity was assessed in a counter-screen on human epithelial colorectal adenocarcinoma cells. MTZ was used as a positive control in the assays. All the tested compounds proved to be more effective against the parasite in the presence of O_2_, with the exception of MTZ that was less effective. Under anaerobiosis eighteen compounds were found to be as effective as MTZ or more (up to three to fourfold); the same compounds proved to be up to >100-fold more effective than MTZ under microaerobic conditions. Four of them represent potential candidates for the design of novel antigiardial drugs, being highly selective against *Giardia* trophozoites. This study further underlines the importance of taking O_2_ into account when testing novel potential antigiardial compounds.

## Introduction

The amitochondriate protozoon *Giardia intestinalis* is a human parasite, causing extensive morbidity worldwide (Ankarklev et al., 2010). Approximately 6–8% of children and 2% of adults are estimated to be infected in urbanized countries around the world (Craun, 1996). In spite of its recognition as an important human pathogen for a long time, nearly 5,000 people are hospitalized with giardiasis annually in the United States (see (Lengerich et al., 1994; Gardner and Hill, 2001) and references therein). The disease spreads through fecal-oral transmission of the parasite cysts (Adam, 2001). The host is typically infected through ingestion of cyst-contaminated water or food. After exposure to the acidic environment of the stomach lumen, the cyst develops into the trophozoite, the vegetative form of the parasite, that in turn attaches to the mucosa of the proximal small intestine. This is the crucial step in establishing and maintaining the infection. In the small intestine, the trophozoite starts proliferating, causing symptoms like diarrhea, malabsorption, dehydration, weight loss, failure to thrive, and chronic fatigue. Following encystation, the parasite is ready to be expelled into the environment and infect a new host, thus completing the life cycle.

Giardiasis is commonly treated with several approved medications, that include metronidazole (MTZ), tinidazole, furazolidone, albendazole, paromomycin, or nitazoxanide (Ali and Nozaki, 2007; Tejman-Yarden and Eckmann, 2011). This notwithstanding, because of the limited efficacy, heavy side effects, and increasing resistance of the parasite to available treatments, it is mandatory to continue searching for novel antigiardial drug candidates (Upcroft and Upcroft, 2001; Ali and Nozaki, 2007). The gold standard drug against giardiasis is MTZ, (Edwards, 1993), a pro-drug that needs to be activated intracellularly by reduction of the nitro moiety (Edwards, 1993). Relevant to this study, by reaction with O_2_ the active form of MTZ is converted back to the inactive parent compound. Presence of O_2_ in the epithelium of the proximal small intestine (Espey, 2013), where *Giardia* trophozoites adhere with their ventral disks, is therefore expected to significantly decrease the efficacy of MTZ *in vivo*. Given the fairly aerobic environment inhabited by *Giardia* in the host, it is important to consider possible effects of O_2_, when testing novel potential antigiardial drugs. Following this rationale, we have recently carried out a study (Bahadur et al., 2014) in which a set of synthetic compounds has been initially screened for its antigiardial activity under anaerobic condition, and then the compounds with the highest activity were assayed for their efficacy under microaerobic conditions too. This innovative approach allowed us to identify two chalcone derivatives that under microaerobic conditions proved to be selectively active against *Giardia* trophozoites and more effective than MTZ (Bahadur et al., 2014).

Nowadays, molecular hybridization is a coherent strategy that allows one to design new chemical entities of potential biomedical relevance by fusing two or more recognized active compounds and/or pharmacophoric units of known bioactive molecules (Viegas-Junior et al., 2007; Walsh and Bell, 2009). In this regard, chalcone derivatives linked to a triazolyl-quinolone moiety represent an attractive drug scaffold. Nitrogen containing heterocycles are indeed widely used for the synthesis of compounds of pharmaceutical interest (Syam et al., 2012) and chalcone analogs with their relatively simple structure have a wide variety of pharmacological activities, largely attributed to their α,β unsaturated ketone moiety (Kumar et al., 2003). Moreover, quinoline (1-azanaphthalene) compounds are widely used as “parental” compounds to synthesize molecules with a broad range of biological activities including anti-inflammatory (el-Gazzar et al., 2009), antileishmanial (Palit et al., 2009), antifungal (Kategaonkar et al., 2010), and antituberculosis (Eswaran et al., 2010). Finally, tiazoles represent another important class of heterocycles because of their varied biological activities and, accordingly, triazole-containing ring systems are found in numerous existing drugs, like fluconazole, itraconazole, and voriconazole, commonly used as anti-inflammatories, CNS-stimulants, sedatives, antianxiety, antimicrobials, and antimycotics (Shaker, 2006; Saadeh et al., 2010).

Here, we have synthesized, purified and thoroughly characterized a set of 45 novel chalcone analogs with triazolyl-quinolone scaffold, and comparatively evaluated their antigiardial activity both in anaerobiosis and microaerobiosis. This led to the identification of four compounds poorly toxic against human cells, yet able to affect *Giardia* trophozoites more effectively than MTZ under both anaerobic and microaerobic conditions.

## Materials and Methods

### Materials

All chemicals used in the synthesis of chalcones analogs were purchased from Sigma–Aldrich and Fluka and were used as such without any prior purification. MTZ, ATP, penicillin/streptomycin, bovine calf serum, bovine bile, and the chemicals for the Diamond’s TYI-S-33 medium used for *Giardia* cell cultures were purchased from Sigma–Aldrich. Fetal bovine serum, glutamine, non-essential amino acids, trypsin-EDTA, and the Eagle’s Minimum Essential Medium (EMEM) were purchased from GIBCO (Life technologies). Other chemicals and solvents purchased locally were of analytical grade. Caco-2 cells (ATCC^®^ HTB-37^TM^) were purchased from Sigma–Aldrich. Incubation bags for anaerobiosis (Anaerocult^®^ A minisystem) and microaerobiosis (Anaerocult^®^ C minisystem) were from Merck. Sterile 96-well white clear-bottom plates were purchased from Perkin Elmer. The ATP one-step luminescence assay systems for microbial (BacTiter-Glo^TM^) and human (ATPlite^TM^) cells were from Promega and Perkin Elmer, respectively.

### Synthesis and Characterization of Chalcones

#### Chemical Methods

Homogeneity/purity of all the products was analyzed by thin-layer chromatography (TLC) on alumina coated plates (Merck). Product samples in MeOH were loaded on TLC plates and developed in CHCl_3_–MeOH (9.8:0.2, v/v). On detection of slight impurities by iodine vapor/UV light visualization, compounds were further purified by chromatography on silica gel columns (100–200 mesh size, CDH), using petroleum ether-ethyl acetate (3:2, v/v) as the eluent. Melting points were determined in open glass capillary tubes on a Buchi M-560 instrument and are uncorrected. Infrared (IR) spectra were recorded in KBr medium using a Perkin-Elmer Fourier Transform-IR spectrometer, whereas ^1^H and ^13^C nuclear magnetic resonance (NMR) spectra were recorded in CDCl_3_ medium on a JNM ECX-400P (JEOL, USA) spectrometer with tetramethylsilane (TMS) as internal reference. IR and NMR spectra were recorded at the Department of Chemistry, University of Delhi, India. Absorption frequencies (ν) are expressed in cm^-1^, chemical shifts in ppm (δ-scale) and coupling constants (J) in Hz. Splitting patterns are described as singlet (s), doublet (d), triplet (t), quartet (q), and multiplet (m). High resolution mass spectroscopy (HRMS) data were collected with a resolution of 10,000 on a KRATOS MS50TC spectrometer and a Kratos Mach III type at the University of Leuven (KU Leuven, Celestijnenlaan 200F, 3001 Leuven, Belgium).

#### General Procedure for the Synthesis of 2-chloroquinoline-3-carbaldehyde (Ramesh et al., 2008) (2)

2-chloroquinoline-3-carbaldehyde was synthesized from acetanilide (**1**) via a Vilsmeier–Haack reaction by traditional methods. To a well-stirred mixture of *N,N*-dimethylformamide (DMF, 3 equiv.) and acetanilide **1** (1 equiv.), POCl_3_ (12 equiv.) was added dropwise slowly at 0^∘^C. Afterward the reaction mixture was heated to 100^∘^C for 16 h and the reaction progress was monitored by TLC. After completion of the reaction, the mixture was allowed to cool down to room temperature and poured into crushed ice under vigorous stirring. The obtained precipitate was filtered, washed with water and re-crystallized from dry EtOH to give the title compound **2** with 62% yield; mp 146–147^∘^C (lit 148^∘^C).

#### General Procedure for the Synthesis of 2-oxoquinoline-3-carbaldehyde (Ramesh et al., 2008) (3)

2-chloroquinoline-3-carbaldehyde **2** was refluxed in 70% acetic acid to obtain 2-oxoquinoline-3-carbaldehyde **3**. The reaction mixture was heated to 110^∘^C for 12 h and the reaction progress was monitored by TLC. After completion of the reaction, the mixture was allowed to cool down to room temperature and poured into crushed ice under vigorous stirring. The obtained precipitate was filtered, washed with water and re-crystallized from dry EtOH to give the title compound **3** with 90% yield; mp 301–302^∘^C (lit 304^∘^C).

#### General Procedure for the Synthesis of 2-oxo-1-(prop-2-ynyl)-1,2-dihydroquinoline-3-carbaldehyde (Pal et al., 2011) (4)

To a solution of 2-oxo-1,2-dihydroquinoline-3-carbaldehyde **3** (1.0 equiv.) in K_2_CO_3_ (1.5 equiv.) and DMF, propargyl bromide (1.5 equiv) was added dropwise. The reaction mixture was stirred at room temperature for 12 h. After completion of the reaction, the mixture was poured into ice-cooled water. The solid separated was filtered, washed, dried, and re-crystallized from ethanol to give the title compound **4** with 75% yield; mp 195–197^∘^C (lit 198^∘^C).

#### General Procedure for the Synthesis of Azidobenzene and its Derivatives (Haridas et al., 2011) (5–9)

To a mixture of appropriate aniline (1 equiv.) in 17% HCl stirred at 0^∘^C, aqueous sodium nitrite (1.2 equiv.) was added dropwise with continued stirring for 10 min at 0^∘^C. Afterward, aqueous sodium azide (1.2 equiv.) was added dropwise to the reaction mixture at 0^∘^C with continued stirring for additional 3 h at room temperature. The reaction progress was monitored by TLC [petroleum ether/ethyl acetate (5:1)]. After completion of the reaction, the mixture was subjected to extraction with ethyl acetate (2 × 25 mL). The combined organic layer was dried over Na_2_SO_4_ and concentrated under reduced pressure, to obtain an oily brown colored compound.

#### General Procedure for the Synthesis of 2-oxo-1-((1-phenyl-1H-1,2,3-triazol-4-yl)methyl)-1,2-dihydroquinoline-3-carbaldehyde (10–14)

The mixture of alkyne **4** (1 equiv.), appropriate azide (**5–9**; 1.5 equiv.), CuSO_4_⋅5H_2_O (0.2 equiv.), and sodium ascorbate (0.4 equiv.) was taken in a (3:1) mixture of tetrahydrofuran (THF) and water and stirred at room temperature for 12 h. The reaction progress was monitored by TLC using CHCl_3_: MeOH (9.7:0.3, v/v) as the solvent system. After completion of the reaction, the mixture was subjected to extraction with ethyl acetate (3 × 30 mL). The combined ethyl acetate layer was dried over Na_2_SO_4_ concentrated under reduced pressure and finally purified by silica gel (100–200 mesh size) column chromatography using petroleum ether – ethyl acetate (3:2) as the eluent to yield the desired product.

#### General Procedure for the Synthesis of (E)-3-(3-oxo-3-phenylprop-1-en-1-yl)-1-((1-phenyl-1*H*-1,2,3-triazol-4-yl)methyl)quinolin-2(1*H*)-one (23–67).

To a solution of aryl ketone (**15–22**; 1 equiv.) and triazolyl-quinaldehyde (**10–14**; 1 equiv.) in dry MeOH, cat. sodium hydroxide was added. The resulting reaction mixture was stirred at room temperature for 24 h. The reaction progress was monitored by TLC using CHCl_3_:MeOH (9.8:0.2, v/v) as the solvent system. After completion of the reaction, the mixture was poured into ice-cool water. The obtained colored precipitate was filtered and dried on vacuum. The compounds were finally purified by silica gel (100–200 mesh size) column chromatography using petroleum ether – ethyl acetate (3:2) as the eluent.

### Assays on Cells

#### Cell Cultures

Trophozoites of *G. intestinalis* strain WB clone C6 (ATCC No. 50803TM) were cultured axenically at 37^∘^C in 25-cm^2^ flasks containing Diamond’s TYI-S-33 medium supplemented with 10% bovine calf serum, 1 mg/mL bovine bile, 0.1 g/L streptomycin and 100 U/mL penicillin. Typically, 50 ml medium was inoculated with 25 × 10^6^ cells and after 2 days cells were harvested by chilling the flasks on ice for 30 min for drug susceptibility assays. Human epithelial colorectal adenocarcinoma (Caco-2) cells were grown in 25-cm^2^ flasks in EMEM supplemented with 1% (v/v) non-essential amino acids, 2 mM glutamine, 5% (v/v) fetal bovine serum, 0.1 g/L streptomycin, and 100 U/mL penicillin.

#### Assays on Giardia trophozoites

The assays were performed following the procedure described in Bahadur et al. (2014), using sterile 96-well white clear-bottom plates. In each well, 50 μL of *Giardia* trophozoites at a density of 1 × 10^5^ cells/mL was added to 50 μL medium containing either the compound to be tested, serially diluted from a stock solution in dimethyl sulfoxide (DMSO), or the same amount of DMSO as a control; this yielded a final density of 5.000 cells/100 μL in each well. Each drug concentration was tested in at least six replicates. MTZ was used as an internal positive control in the assay. The microtiter plates were then incubated at 37^∘^C under anaerobic or microaerobic conditions, ensured by the Anaerocult^®^ A or the Anaerocult^®^ C minisystem (Merck), respectively. According to the manufacturer instructions, the Anaerocult^®^ A minisystem produces anaerobic conditions within ∼1 h, whereas the Anaerocult^®^ C minisystem generates microaerobic conditions (∼5% O_2_) within 24 h. Following 48 h incubation with the compound to be tested, 100 μL of the BacTiter-Glo^TM^ Microbial Cell Viability Assay System reagent (Promega) was added to each well for one-step lysis and ATP level detection. Plates were then incubated at room temperature for 15 min and ATP levels finally detected by luminescence on a plate reader (Wallac Victor3 1420 Multilabel Counter, PerkinElmer).

#### Assays on Caco-2 Cells

Cells were detached with 0.5% trypsin-EDTA and seeded in sterile 96-well white clear-bottom plates at the same density of *Giardia* trophozoites and at increasing concentration of the compound to be tested, as described above. The assays were carried out exactly as reported for the *Giardia* trophozoites, except that the plates were incubated (still at 37^∘^C) at atmospheric O_2_ level, 5% CO_2_, and 95% humidity. Each drug concentration was tested in at least seven replicates. MTZ was used as an internal negative control in the assay. Following 48 h incubation with each compound, according to the manufacturer instructions, 100 μL of the ATPlite^TM^ luminescence assay system (Perkin Elmer) was added to each well for one-step lysis and ATP level detection by luminescence.

#### Determination of Half-Maximal Inhibitory Concentration (IC_50_) and Selectivity Index (SI)

Luminometric data were calibrated using ATP standard curves and normalized to the ATP level measured in control DMSO-treated cells (taken as 100%). The measured ATP level percentage was plotted as a function of the compound concentration and the half-maximal inhibitory concentration (IC_50_) was obtained by fitting the resulting titration profile to the Hill equation (Goutelle et al., 2008). The selectivity index (SI) of the compounds was then calculated as the ratio between the IC_50_ value measured on human cells over the value determined on *Giardia* trophozoites (SI = IC_50,Caco-2_/IC_50,Giardia_).

## Results

### Synthesis of Novel Triazolyl-Quinolone Based Chalcones

**FIGURE 1:**
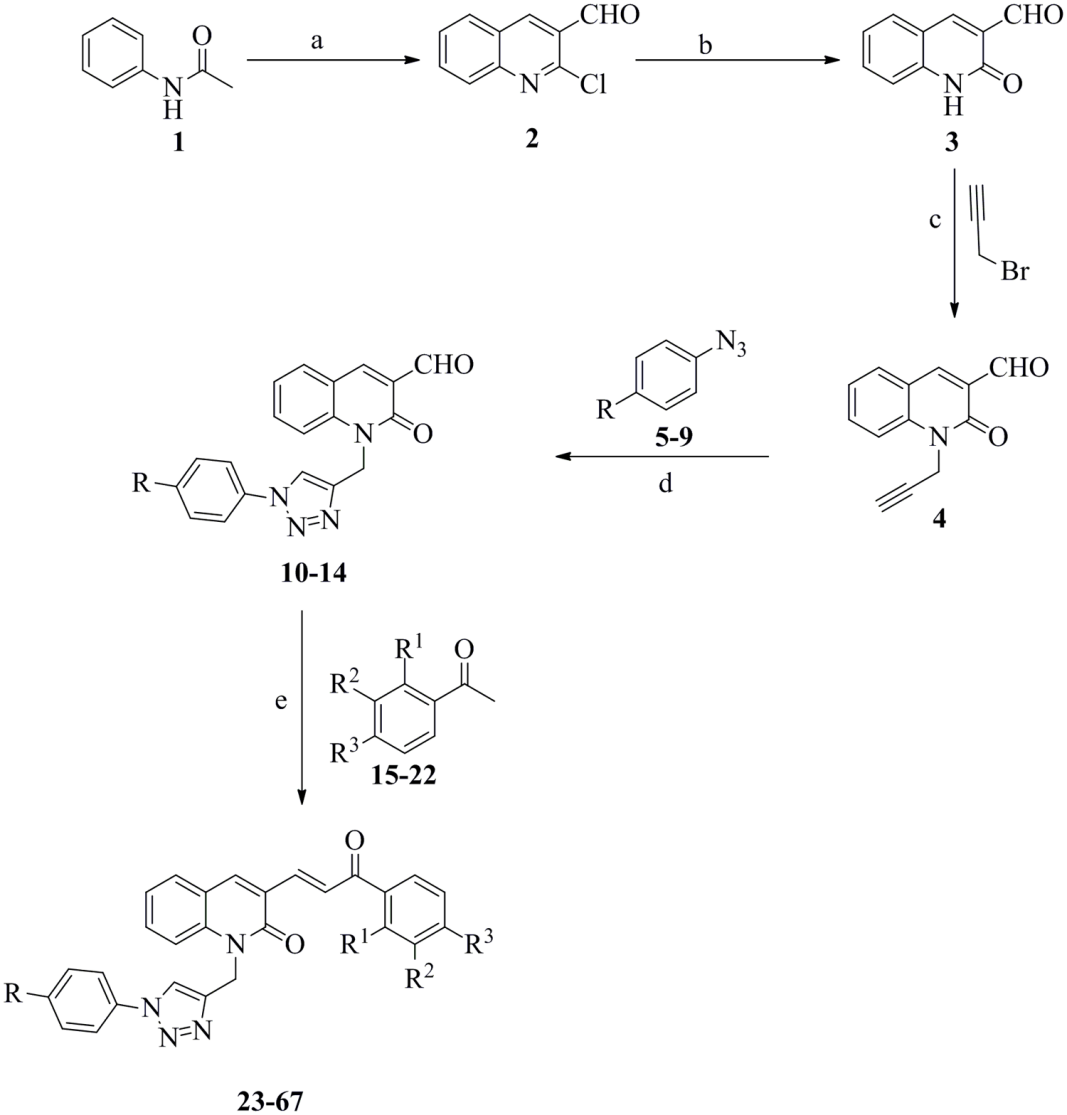
**Synthetic route to compounds 23–67.** (a) DMF, POCl_3_, 0–100^∘^C, 16 h; (b) 70% acetic acid, 110^∘^C, 12 h; (c) K_2_CO_3_, DMF, rt, 12 h; (d) CuSO_4_⋅5H_2_O, Na-ascorbate, THF:H_2_O (3:1), rt, 12 h; (e) NaOH, MeOH, rt, 24 h.

Forty-five novel triazolyl-quinolone-based chalcone derivatives were synthesized based on previously described Claisen-Schmidt condensation (Li et al., 1995), according to the synthetic route outlined in **Figure 1**. Intermediate triazolyl-quinolone compounds **10–14** were synthesized at room temperature in THF:water with 50–75% yield, by “click chemistry” (Barral et al., 2007) of the synthesized alkyne **4** with appropriate aromatic azides (**5–9**), using CuSO_4_⋅5H_2_O and sodium ascorbate as catalysts. As shown in **Figure 1**, compounds **23–67** were obtained at room temperature by reaction of the intermediate compounds **10–14** with commercially available aromatic acetophenones **15–22**, in the presence of NaOH in dry MeOH. After purification, the yield of products **23–67** ranged from 60 to 95%.

The newly synthesized triazolyl-quinolone-based chalcone derivatives were characterized by HRMS, ^1^H and ^13^C NMR and IR spectroscopy, and relevant data are reported in Supplementary Material.

### Antigiardial Activity of the Synthesized Chalcones

**FIGURE 2:**
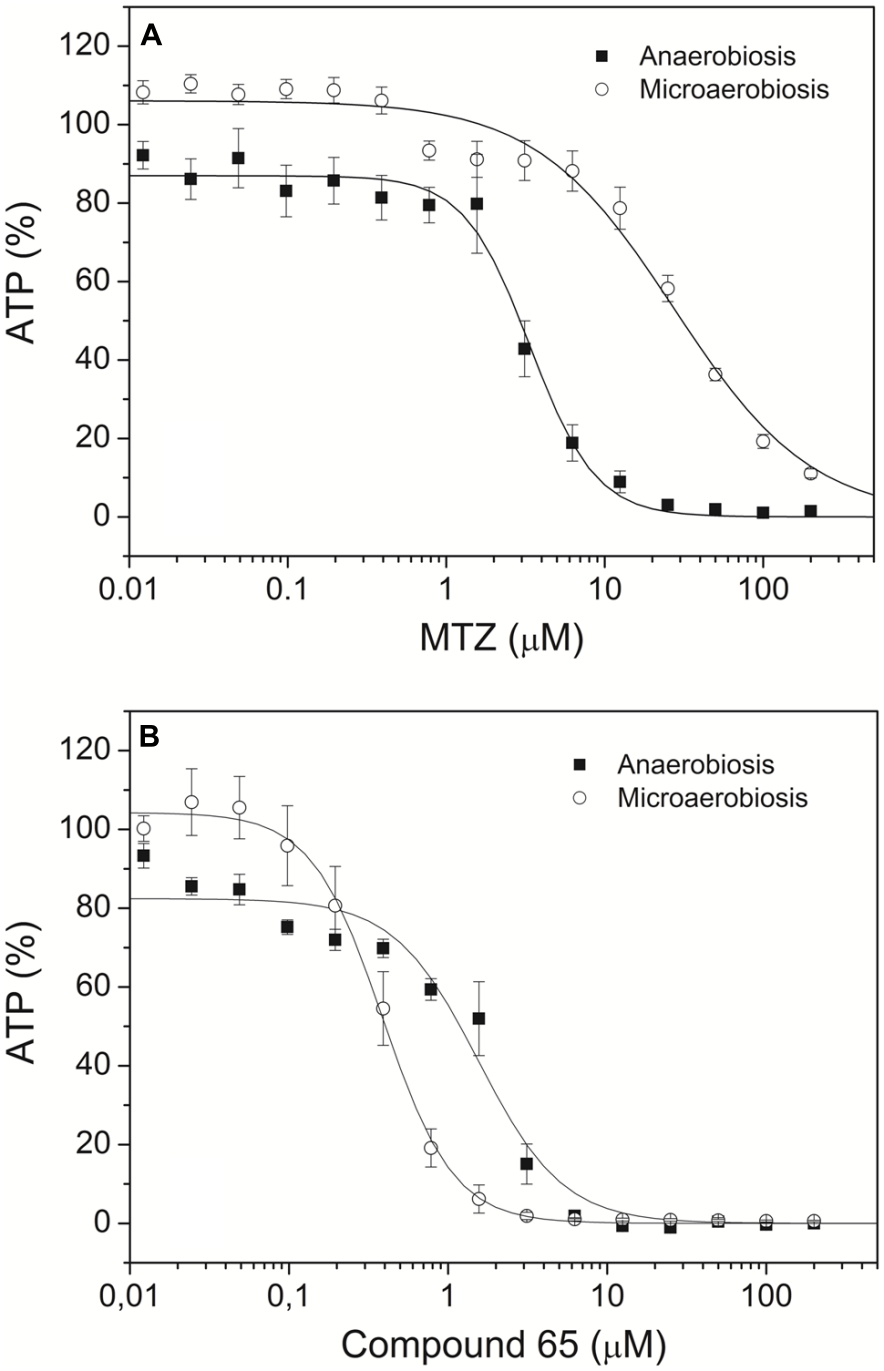
**Effect of metronidazole (MTZ) and compound 65 on *Giardia* trophozoites.** Dose-response curves of MTZ **(A)** and the newly synthesized compound **65** **(B)**, measured on *Giardia* trophozoites cultured under anaerobic (closed symbol) or microaerobic (open symbol) conditions. Data are expressed as mean ± SEM (*n* ≥ 6).

The antigiardial activity of the novel compounds **23–67** was tested under both anaerobic and microaerobic conditions. According to (Dunn et al., 2010; Bahadur et al., 2014), susceptibility of *Giardia* trophozoites to increasing concentrations of each compound was assessed based on ATP level determination by luminescence. Dose-response curves for each compound were obtained after 48 h-incubation and compared to the data collected under identical conditions with MTZ, the drug of choice for treatment of giardiasis. In these assays, the ATP level measured in control *Giardia* trophozoites grown under microaerobic conditions was found to be approximately 25% lower than in the same cells grown under anaerobic conditions, and DMSO at concentrations ≤2%_v/v_ caused only marginal effects on cell ATP levels. Typical dose-response curves are shown in **Figure 2**, whereas the IC_50_ values measured for the synthetic compounds and MTZ under anaerobic and microaerobic conditions are reported in **Table 1**. In agreement with the literature (Müller et al., 2006), under the experimental conditions of the assay, MTZ proved to be highly effective (IC_50_ = 3.4 μM) against *Giardia* parasites in the absence of O_2,_ but remarkably less (IC_50_ ≥ 25 μM) under microaerobic conditions. Under anaerobic conditions, 18 out of the 45 synthetic compounds were as effective as MTZ or more under identical conditions (see **Table 1**). Among them, compounds **41**, **43,** and **45** displayed the highest activity, being three to fourfold more efficient than MTZ.

**Table 1:** Chemical identity, IC_50_ values and selectivity index (SI) of the novel synthetic compounds.

					***Giardia *(anaerobiosis)**	***Giardia *(microaerobiosis)**	**Caco-2**	**Selectivity (IC_50,Caco_/IC_50,Giardia_**)
**S. No**	**R**	**R^1^**	**R^2^**	**R^3^**	**IC_50_**	**IC_50_**	**IC_50_**	**Anaerobiosis**	**Microaerobiosis**
23	OMe	F	H	H	≥100	≥20	≥100	n.d.	n.d.
24	OMe	H	F	H	≥100	8,4	≥50	n.d.	≥6
25	OMe	H	H	F	3,9	1,3	≥20	≥ 5	≥15
26	OMe	H	H	Cl	5,0	2,6	≥20	≥4	≥7
27	OMe	H	H	Br	9,6	2,0	≥20	≥2	≥10
28	OMe	H	H	Me	5,4	1,5	≥80	≥14	≥53
**29**	**OMe**	**OMe**	**H**	**H**	**2,2**	**0,4**	**≥40**	**≥18**	**≥100**
30	OMe	H	OMe	H	5,9	1,3	≥200	≥33	≥153
31	OMe	H	H	OMe	≥50	2,0	≥80	n.d.	≥40
**32**	**Me**	**F**	**H**	**H**	**3,2**	**0,6**	**17,6**	**5,5**	**29,3**
33	Me	H	F	H	4,0	0,7	13,4	3,4	19,1
34	Me	H	H	F	3,8	1,2	9,4	2.5	7.8
**35**	**Me**	**H**	**H**	**Cl**	**2,4**	**0,8**	**7,0**	**2.9**	**8.8**
36	Me	H	H	Br	10,7	2,3	≥50	≥4	≥21
37	Me	H	H	Me	6,3	1,6	≥50	≥7	≥31
**38**	**Me**	**OMe**	**H**	**H**	**2,0**	**0,4**	**13,7**	**6,9**	**34,3**
**39**	**Me**	**H**	**OMe**	**H**	**2,9**	**1,3**	**≥100**	**≥34**	**≥76**
40	Me	H	H	OMe	≥15	2,4	≥60	n.d.	≥25
**41**	**F**	**F**	**H**	**H**	**0,9**	**0,2**	**5,2**	**5,8**	**26**
**42**	**F**	**H**	**F**	**H**	**3,3**	**0,7**	**9,6**	**2,9**	**13,7**
**43**	**F**	**H**	**H**	**F**	**1,2**	**0,3**	**7,0**	**5,8**	**23,3**
**44**	**F**	**H**	**H**	**Cl**	**1,5**	**1,0**	**7,2**	**4.8**	**7,2**
**45**	**F**	**H**	**H**	**Br**	**1,1**	**0,5**	**16,7**	**15,2**	**33,4**
**46**	**F**	**H**	**H**	**Me**	**2,5**	**0,4**	**6,3**	**2,5**	**15,8**
**47**	**F**	**OMe**	**H**	**H**	**2,7**	**0,3**	**≥20**	**≥7**	**≥66**
48	F	H	OMe	H	7,0	1,6	≥ 60	≥8	≥37
49	F	H	H	OMe	13,0	1,3	≥50	≥3	≥38
50	Cl	F	H	H	4,7	0,9	≥ 50	≥10	≥55
51	Cl	H	F	H	10,0	0,9	≥100	≥10	≥111
52	Cl	H	H	F	8,9	1,2	23,9	2,7	19,9
53	Cl	H	H	Cl	9,0	1,1	≥30	≥3	≥27
54	Cl	H	H	Br	9,2	1,9	≥20	≥ 2	≥10
55	Cl	H	H	Me	8,7	1,1	20	2,3	18,2
**56**	**Cl**	**OMe**	**H**	**H**	**3,2**	**0,6**	**8,4**	**2,6**	**14,0**
57	Cl	H	OMe	H	6,6	1,1	14,7	2,2	11,3
58	Cl	H	H	OMe	≥100	≥30	19,7	≤0,2	≤0,7
**59**	**H**	**F**	**H**	**H**	**1,9**	**0,4**	**11,2**	**5,9**	**28,0**
60	H	H	F	H	3,5	0,7	≥50	≥14	≥71
61	H	H	H	F	5,3	0,8	18,2	3,4	22,8
**62**	**H**	**H**	**H**	**Cl**	**2,1**	**0,7**	**5,9**	**2.8**	**8,4**
63	H	H	H	Br	6,3	1,0	≥50	≥ 7	≥ 50
**64**	**H**	**H**	**H**	**Me**	**2,8**	**0,5**	**17,8**	**6,4**	**35,6**
**65**	**H**	**OMe**	**H**	**H**	**1,6**	**0,4**	**≥50**	**≥31**	**≥125**
**66**	**H**	**H**	**OMe**	**H**	**3,2**	**1,6**	**≥50**	**≥15**	**≥31**
67	H	H	H	OMe	5,9	0,8	≥100	≥16	≥125
**MTZ**					**3,4**	**≥25**	**≥100**	**≥29.0**	**n.d.**

**Bold, **IC_50_ < IC_50,MTZ_ in anaerobiosis; Compounds **29**, **39**, **65** and **66** display IC_50_ < IC_50,MTZ_ in both anaerobiosis and microaerobiosis, low toxicity against Caco-2 cells (IC_50_ ≥ 40.0) and SI = 10.

Interestingly, at variance from MTZ, all the tested compounds displayed a higher efficacy against *Giardia* under microaerobic conditions than in the absence of O_2_. Notably, apart from a few exceptions (namely, compounds **23** and **58**), due to their enhanced efficacy and the notably lower activity of MTZ, all the screened compounds were found to be more effective than MTZ under microaerobic conditions. In many cases, the effect of O_2_ was remarkable, as it can be seen from the IC_50_ values reported in **Table 1**. Under microaerobic conditions approximately half of the tested compounds proved to be at least fivefold more active against *Giardia* than in the absence of O_2_. The effect of O_2_ was particularly evident in the case of compounds **24,** **31**, **49,** and **51**, which displayed ≥10-fold higher activities under microaerobic conditions than measured in anaerobiosis. Interestingly, up to 10 compounds displayed IC_50_ ≤ 0.5 μM under microaerobic conditions, thereby becoming >50-fold more active than MTZ under identical conditions.

### Selectivity of the Synthetic Compounds

Selectivity of the newly synthesized compounds toward *Giardia* was assessed by running a counter-screen on human epithelial Caco-2 cells. Similarly to the screen carried out on *Giardia* trophozoites, dose-response curves and corresponding IC_50_ values for each compound were obtained after 48 h-incubation, as reported above. This allowed us to calculate the compounds SI, defined as the ratio between the IC_50_ value measured on human cells over the value determined on *Giardia* trophozoites. In the assays on human cells, DMSO affected ATP cell levels only slightly (up to 10% at ≤ 2%v/v, not shown) and, as expected, MTZ exhibited a high IC_50_ value (>100 μM). Interestingly, under the same experimental conditions, 19 compounds were only poorly effective against human cells, being characterized by IC_50_ values≥40 μM (**Table 1**). Among these, compounds **29**, **39**, **65,** and **66** were particularly interesting in that they proved to be (i) more effective than MTZ against *Giardia* trophozoites both in anaerobiosis (IC_50_ ranging from 2.2 to 3.2 μM) and microaerobiosis (IC_50_ ranging from 0.4 to 1.6 μM), and (ii) much less effective on human cells (IC_50_ ≥ 40 μM), thereby exhibiting a high selectivity toward *Giardia* trophozoites (with SI values exceeding 15 and 30 under anaerobiosis and microaerobiosis, respectively).

## Discussion

Compared to other more distal tracts of the gut, the proximal small intestine is a fairly aerobic environment, where O_2_ is supplied mostly by the submucosal vascular network and in part with swallowed air (Dawson et al., 1965; Levitt, 1970; Sheridan et al., 1990; He et al., 1999). In this tract of the gut, O_2_ concentration is relatively high and subjected to sudden oscillations, particularly in the postprandial period when the metabolic demand increases upon transit of partly processed food (Espey, 2013). Relevant to the present study, O_2_ concentration is particularly high at the level of the intestinal epithelium, where trophozoites of *G. intestinalis* adhere with their ventral disks. O_2_ tension indeed decreases along a steep gradient from 80–100 to nearly 0 mm Hg, moving inward from the submucosa toward the luminal midpoint (Espey, 2013). Therefore, though commonly regarded as an anaerobic protozoon, *Giardia* is likely exposed to fairly high and variable O_2_ levels *in vivo*. The parasite is amitochondriate and thus unable to utilize O_2_ to sustain energy metabolism. This notwithstanding, μM O_2_ concentrations have been shown to produce profound changes in the metabolism of *Giardia* trophozoites, leading to stimulation of both ethanol and CO_2_ production, elicited oxidation of the intracellular NAD(P)H pool and reduced alanine production (Paget et al., 1993). In addition to these adaptive metabolic changes, in order to survive oxidative stress conditions, *Giardia* needs to activate the antioxidant defense system (Brown et al., 1995; Mastronicola et al., 2011; Raj et al., 2014). In this regard, it has been established that, though lacking *bona fide* catalases or superoxide dismutases, *Giardia* is endowed with several alternative antioxidant enzymes that have been recently identified and partly characterized. These include: a NADH oxidase (Brown et al., 1996a) and a flavodiiron protein (Di Matteo et al., 2008; Vicente et al., 2009) detoxifying O_2_ to H_2_O, a flavohemoglobin converting nitric oxide into nitrate (Mastronicola et al., 2010, 2011; Rafferty et al., 2010), a superoxide reductase promptly degrading superoxide anion to H_2_O_2_ (Testa et al., 2011), a thioredoxin reductase (Brown et al., 1996b) and two peroxiredoxins (Mastronicola et al., 2014) implicated in peroxide detoxification and repair of oxidatively damaged molecules. Exposure to O_2_ is therefore expected to cause notable changes in the metabolic and proteomic profile of *G. intestinalis*, an aspect that has not been studied in detail thus far.

Despite being physiologically exposed to O_2_ *in vivo*, *Giardia* trophozoites are commonly assayed *in vitro* for their drug susceptibility under anaerobic conditions. This may bias the results of a screening. Leading to changes in cell metabolism, O_2_ may indeed modify the susceptibility of the parasite to specific drugs, possibly up- or down-regulating molecular targets. Moreover, by directly reacting with the tested drugs, it may alter their mechanism of action, possibly leading to enhanced or reduced efficacy. This is the case of MTZ, the gold standard drug against giardiasis, whose efficacy is abolished upon reaction of its nitro-radical active form with O_2_ (Edwards, 1993). When testing new potential antigiardial drugs, it is thus important to take O_2_ into account also to attempt identifying antiparasitic compounds that could be fully effective under the more physiological microaerobic conditions in which MTZ efficacy is reduced due to O_2_ presence. This issue has been highlighted in a recent study by our groups (Bahadur et al., 2014) in which O_2_, while making MTZ expectedly less effective, was reported to enhance the efficacy of three piperidine/piperazine chalcone derivatives, pre-selected in a screen conducted under anaerobic conditions.

In the present study, 45 novel chalcone derivatives with triazolyl-quinolone scaffold were synthesized, purified, and characterized by HRMS, ^1^H and ^13^C NMR and IR spectroscopy. As an innovative approach, all the compounds were comparatively tested for their efficacy against *G. intestinalis* trophozoites under both anaerobic and microaerobic conditions. Compared to anaerobic conditions, the presence of O_2_ was found to increase the IC_50_ of MTZ from 3.4 to ≥25 μM. This result agrees with the notion that O_2_ interferes with MTZ, by converting its nitro-radical active form into the parental inactive form (Edwards, 1993). However, noteworthy, in spite of the reduced efficacy of MTZ, all the tested compounds proved to be more effective in the presence of O_2_, though to different extent. For most of the compounds the IC_50_ was four to sevenfold smaller than measured under anaerobic conditions, but in the case of four compounds (**24,** **31**, **49,** and **51**) O_2_ was particularly effective decreasing their IC_50_ by more than 10-fold. While under anaerobic conditions only 18 out of the 45 tested compounds proved to be as active as MTZ or more, in the presence of O_2_ almost all the tested compounds (with the exception of compounds **23** and **58**) were more effective than MTZ. Under microaerobic conditions, up to 10 compounds showed notably low IC_50_ values (≤0.5 μM), thereby becoming >50-fold more effective than MTZ under the same microaerobic conditions, yet less effective than the alternative antigiardial drug albendazole that under anaerobic conditions shows very low IC_50_ values (<0.1 μM, Cedillo-Rivera and Munoz, 1992). Interestingly, 19 out of the 45 tested compounds displayed very poor toxicity against human Caco-2 cells (IC_50_≥40 μM), and by comparing their efficacy on human and *Giardia* cells 10 out of these 19 compounds proved to be highly selective toward the parasite, being ≥10-fold more effective against *Giardia* under both anaerobic and microaerobic conditions. Among these 10 compounds highly selective against *Giardia*, four can be considered as hits to develop potential antigiardial drugs, namely compounds **29**, **39**, **65**, and **66**, being more effective than MTZ under both anaerobic and aerobic conditions.

The compounds characterized here carry substitutions in two phenyl rings: ring **A** (attached to the triazole moiety) and ring **B** (attached to the α,β unsaturated moiety). By inspecting , it can be noted that on average the compounds with the highest antigiardial activity have either unsubstituted (compounds **59**–**67**) or fluoro-substituted (compounds **41–49**) ring **A**. Comparatively, less antigiardial activity is observed when the same ring is substituted with chloro (compounds **50–58**), methoxy (compounds **23**–**31**) or methyl (compounds **32–40**) groups. Compared to other types of substitutions in ring **A**, the fluoro-substitution seems to result into higher toxicity of the compounds against human cells. The occurrence of substitutions at ring **B** and their position in the ring also appear to modulate the antigiardial activity. Comparison of compounds **29**, **30,** and **31** shows that the position of a methoxy substituent in ring **B** has great effects on the antiparasitic activity of the molecules. The *ortho* substituted compound **29** is more effective than the *meta* substituted compound **30**, that in turn displays a higher antigiardial activity compared to the *para* substituted analog (compound **31**). Interestingly, the same trend (*ortho* > *meta* > *para*) is invariably observed by comparing the compound triplets **38–40**, **47–49**, **56–58,** and **65–67**, all having methoxy-substituted ring **B**. Finally, it is worth noticing that the compounds optimally combining high antigiardial activity and low toxicity against human cells (compounds **29**, **39**, **65**, and **66**) have ring **B** invariably methoxy-substituted at *ortho* or *meta* positions, and ring **A** either unsubstituted or substituted with methoxy or methyl groups.

To the best of our knowledge, this is the first study in which a complete set of new synthetic compounds has been screened for its ability to affect *Giardia* trophozoites under both anaerobic and microaerobic conditions, providing unequivocal evidence for an effect of O_2_. The results show that, as compared to anaerobic conditions, the presence of low, more physiological O_2_ concentrations elicits the antiparasitic activity of the tested compounds, while having opposite effects on MTZ. The molecular mechanism underlying the observed effects of O_2_ has yet to be established. A possibility is that O_2_, by directly affecting *Giardia* trophozoites, on the one hand impairs MTZ activation and on the other makes the cells more susceptible to the novel synthetic drugs here described. A more intriguing possibility is that, in response to O_2_ exposure, the parasite activates specific metabolic pathways that are selectively targeted by the compounds tested in the present study, but not MTZ. To address this issue, it would important in future studies to acquire more detailed information on the pathways that are activated in *Giardia* in the metabolic transition from anaerobic to microaerobic conditions.

## Conclusion

In this study we have identified four new compounds that under both anaerobic and more physiological microaerobic conditions are highly effective against *Giardia* trophozoites, targeting the parasite selectively and more efficiently than MTZ. These four synthetic chalcone derivatives represent potential candidates for the design of novel antigiardial drugs. This work further highlights that it is important to take O_2_ into account when screening new potential antigiardial drugs.

## Conflict of Interest Statement

The authors declare that the research was conducted in the absence of any commercial or financial relationships that could be construed as a potential conflict of interest.
