# Supplementary material for: Antigiardial activity of novel triazolyl-quinolone-based chalcone derivatives: when oxygen makes the difference
Source: Front Microbiol. 2015 Apr 8;6:256. doi: 10.3389/fmicb.2015.00256 (PMC4389562; doi:10.3389/fmicb.2015.00256)
Supplement: Supplementary file 1 [file data_sheet_1.pdf]

February 12, 2015

Supplementary Material

Antigiardial activity of novel triazolyl quinolone-based chalcone derivatives: when oxygen makes the difference

Vijay Bahadur[1,§], Daniela Mastronicola[2,4,§], Amit Kumar Singh[1], Hemandra Kumar Tiwari[1], Leopoldo Paolo Pucillo[3], Paolo Sarti[2,4], Brajendra Kumar Singh[1,*], Alessandro Giuffrè[2,*]

[1]Bio-Organic Laboratory, Department of Chemistry, Delhi University, Delhi, India
[2]CNR Institute of Molecular Biology and Pathology, Rome, Italy
[3]L. Spallanzani National Institute for Infectious Diseases, IRCCS, Rome, Italy
[4]Department of Biochemical Sciences and Istituto Pasteur – Fondazione Cenci Bolognetti, Sapienza University of Rome, Italy

[§]These Authors contributed equally to this work

*** Correspondence:**
- Alessandro Giuffrè, CNR Institute of Molecular Biology and Pathology, Piazzale Aldo Moro 5, I-00185 Rome, Italy, alessandro.giuffre@uniroma1.it
- Brajendra Kumar Singh, Department of Chemistry, University of Delhi, Delhi 110-007, India, singhbk@chemistry.du.ac.in

1. Supplementary Data

Physico-chemical properties of the newly synthesized compounds:

2-oxo-1-((1-phenyl-1H-1,2,3-triazol-4-yl)methyl)-1,2-dihydroquinoline-3-carbaldehyde (**10**)

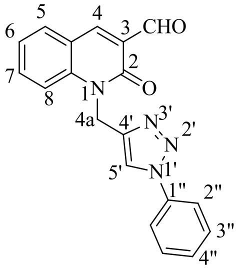

10

Yield: 77%, light brown solid.

Melting Point: 180-182 °C.

IR (KBr) (cm^{-1}): ν 3025 (sp^3 C-H), 1665 (C=O) 1599 (C=C).

^{1}H NMR (400 MHz, $CDCl_3$): δ 5.66 (2H, s, C-4aH), 7.27-7.31 (1H, m, Ar-H), 7.55-7.62 (5H, m, Ar-H), 7.71-7.75 (2H, m, Ar-H), 8.04 (2H, d, *J* = 8.54 Hz, Ar-H), 8.12 (1H, s, C-4H), 8.38 (1H, s, C-5'sH), 10.46(1H, s, C<u>H</u>O).

^{13}C NMR (100 MHz, $CDCl_3$): 38.18 (C-4a), 115.65, 119.44, 120.33, 121.86, 123.41, 125.00, 130.19, 131.83, 134.32, 134.49, 139.03, 141.38, 142.13, 143.45 (C-2), 161.70, 189.73 (<u>C</u>HO).

HRMS: *m/z* Calculated for $C_{19}H_{15}N_4O_2$ $[M+H]^+$ 331.1190 found 331.1205.

1-((1-(4-fluorophenyl)-1*H*-1,2,3-triazol-4-yl)methyl)-2-oxo-1,2-dihydroquinoline-3-carbaldehyde (**11**)

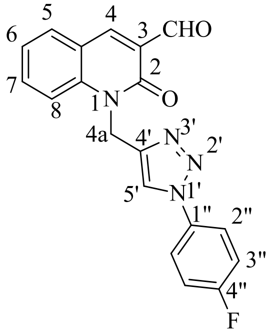

11

Yield: 55%, light yellow solid.

Melting Point: 202-204 °C.

IR (KBr) (cm^{-1}): ν 3022 (sp^3 C-H), 1685 (C=O), 1590 (C=C), 822 (*p*-substitution).

^{1}H NMR (400 MHz, $CDCl_3$): δ 5.88 (2H, s, C-4aH), 7.27-7.33 (1H, m, Ar-H), 7.45-7.49 (1H, m, Ar-H), 7.58-7.66 (3H, m, Ar-H), 7.73-7.80 (2H, m, Ar-H), 7.87-7.93 (2H, m, Ar-H), 8.14 (1H, s, C-4H), 8.63 (1H, s, C-5'H), 10.48 (1H, s, C<u>H</u>O).

^{13}C NMR (100 MHz, $CDCl_3$): δ 37.88 (C-4a), 121.82, 122.04, 125.40, 127.19, 129.85, 132. 81, 132.92, 140.35, 144.33, 159.83 (C -2), 188.87 (CHO).

HRMS: *m/z* Calculated for $C_{19}H_{14}FN_4O_2$ $[M+H]^+$ 349.1095 found 349.1095.

1-((1-(4-chlorophenyl)-1*H*-1,2,3-triazol-4-yl)methyl)-2-oxo-1,2-dihydroquinoline-3-carbaldehyde (**12**)

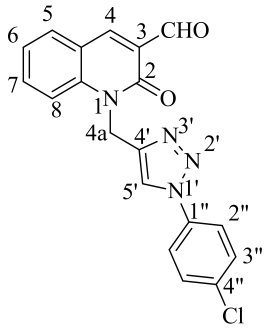

12

Yield: 60%, light brown solid.

Melting Point: 200-202 °C.

IR (KBr) (cm^{-1}): ν 3011 (sp^3 C-H), 1675 (C=O), 1597 (C=C), 830 (*p*-substitution).

^{1}H NMR (400 MHz, $CDCl_3$): δ 5.68 (2H, s, C-4aH), 7.29-7.36 (1H, m, Ar-H), 7.44-7.50 (3H, m, Ar-H), 7.63-7.66 (2H, m, Ar-H), 7.73-7.77 (2H, m, Ar-H), 8.06 (1H, d, *J* = 8.56 Hz, Ar-H), 8.14 (1H, s, C-4H), 8.40 (1H, s, C-5'H), 10.49 (1H, s, -C<u>H</u>O).

^{13}C NMR (100 MHz, $CDCl_3$): δ 38.10 (C-

4a), 115.52, 119.46, 121.60, 121.88, 123.49, 125.00, 129.91, 131.89, 134.36, 134.72, 135.37, 141.46, 142.21, 161.70 (C-2a), 189.64 (<u>C</u>HO).

HRMS: *m/z* Calculated for $C_{19}H_{14}ClN_4O_2$ $[M+H]^+$ 365.0800 found 365.0807.

2-oxo-1-((1-(*p*-tolyl)-1*H*-1,2,3-triazol-4-yl)methyl)-1,2-dihydroquinoline-3-carbaldehyde (13)

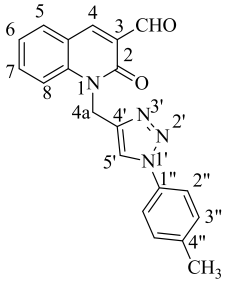

13

Yield: 65%, yellow solid.

Melting Point: 220-222 °C.

IR (KBr) (cm^{-1}): ν 3020 (sp^3 C-H), 1680 (C=O), 1587 (C=C), 812 (*p*-substitution).

^{1}H NMR (400 MHz, $CDCl_3$): δ 2.36 (3H, s, CH_3), 5.66 (2H, s, C-4aH), 7.24-7.30 (3H, m, Ar-H), 7.53 (2H, d, *J* = 8.54 Hz, Ar-H), 7.71 (2H, d, *J* = 7.93 Hz, Ar-H), 8.04-8.08 (2H, m, Ar-H), 8.37 (1H, s, C-5'H), 10.47 (1H, s, C<u>H</u>O).

^{13}C NMR (100 MHz, $CDCl_3$): δ 21.04 (<u>C</u>H_3), 38.16 (C-4a), 115.64, 120.32, 121.87, 123.40, 124.99, 130.18, 131.81, 134.30, 134.48, 139.02, 14 1.36, 142.11, 161.68 (C-2a), 189.71 (<u>C</u>HO).

HRMS: *m/z* Calculated for $C_{20}H_{17}N_4O_2$ $[M+H]^+$ 345.1346 found 345.1340.

1-((1-(4-methoxyphenyl)-1*H*-1,2,3-triazol-4-yl)methyl)-2-oxo-1,2-dihydroquinoline-3-carbaldehyde (14)

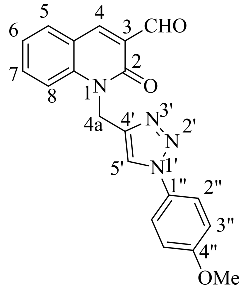

14

Yield: 70%, yellow solid.

Melting Point: 210-212 °C.

IR (KBr) (cm^{-1}): ν 3005 (sp^3 C-H), 1669 (C=O), 1595 (C=C), 825 (*p*-substitution) 1090 (C-O-C).

^{1}H NMR (400 MHz, $CDCl_3$): δ 3.81 (3H, s, OCH_3), 5.65 (2H, s, C-4aH), 6.96 (2H, d, *J* = 9.16 Hz, Ar-H), 7.28-7.30 (1H, m, Ar-H), 7.56 (2H, d, *J* = 9.16 Hz, Ar-H), 7.70-7.74 (2H, m, Ar-H), 8.00 (2H, d, *J* = 9.16 Hz, Ar-H), 8.37 (1H, s, C-5'H), 10.47 (1H, s, C<u>H</u>O).

^{13}C NMR (100 MHz, $CDCl_3$): δ 38.19 (C-

4a), 55.57 (O<u>C</u>H_3), 114.70, 115.65, 119.44, 122.07, 123.41, 125.01, 131.82, 134.32, 14 1.39, 142.13, 159.86 (C-2), 189.73 (<u>C</u>HO).

HRMS: *m/z* Calculated for $C_{20}H_{17}N_4O_3$ $[M+H]^+$ 361.1295 found 361.1302.

(*E*)-3-(3-(2-fluorophenyl)-3-oxoprop-1-en-1-yl)-1-((1-(4-methoxyphenyl)-1*H*-1,2,3-triazol-4-yl)methyl)quinolin-2(1*H*)-one (**23**)

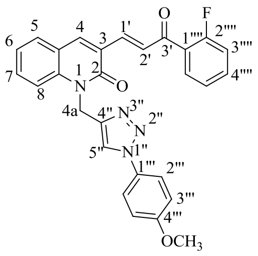

23

Yield: 68%, yellow solid.

Melting Point: 215-217 °C.

IR (KBr) (cm^{-1}): ν 2957 (sp^3 C-H), 1727 (C=O), 1629 (C=C), 1082 (C-O-C), 820 (*p*-substitution).

^{1}H NMR (400 MHz, $CDCl_3$): δ 3.80 (3H, s, -OMe), 5.69 (2H, s, C-4aH), 6.92-6.94 (2H, m, Ar-H), 7.12-7.14 (2H, m, Ar-H), 7.25-7.27 (1H, m, Ar-H), 7.54-7.56 (3H, m, Ar-H), 7.60-7.65 (2H, m, Ar-H), 7.78 (1H, d, *J* = 15.57 Hz, C-2'H), 7.97-8.02 (3H, m, Ar-H), 8.09-8.13 (1H, m, Ar-H), 8.45 ((1H, d, *J* = 15.57 Hz, C-1'H).

^{13}C NMR (100 MHz, $CDCl_3$): δ 38.68 (C-4a), 55.55 (-O<u>C</u>H_3), 111.68, 114.66, 115.28, 115.56, 120.31, 120.33, 121.86, 122.08, 123.02, 125.34, 125.68, 129.64, 130.26, 131.25, 131.34, 132.48, 139.12, 140.05, 142.68, 143.73, 159.80, 160.90 (C-2), 189.28 (C-3').

HRMS: *m/z* Calculated for $C_{28}H_{21}N_4O_3$ $[M]^+$ 480.1598 found 480.1555.

(*E*)-3-(3-(3-fluorophenyl)-3-oxoprop-1-en-1-yl)-1-((1-(4-methoxyphenyl)-1*H*-1,2,3-triazol-4-yl)methyl)quinolin-2(1*H*)-one (**24**)

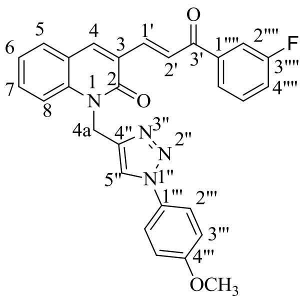

24

Yield: 70%, yellow solid

Melting Point: 210-212 °C.

IR (KBr) (cm^{-1}): ν 2967 (sp^3 C-H), 1720 (C=O), 1625 (C=C), 1080 (C-O-C), 820 (*p*-substitution).

^{1}H NMR (400 MHz, $CDCl_3$): δ 3.75 (3H, s, -OMe), 5.64 (2H, s, C-4aH), 6.87-6.89 (2H, m, Ar-H), 7.19-7.23 (1H, m, Ar-H), 7.38-7.41 (2H, m, Ar-H), 7.50-7.61 (4H, m, Ar-H), 7.74 (1H, d, *J* = 15.11 Hz, C-2'H), 7.93-7.98 (5H, m, Ar-H), 8.39 ((1H, d, *J* = 15.11 Hz, C-1'H).

^{13}C NMR (100 MHz, $CDCl_3$): δ 38.68 (C-4a), 55.54 (-O<u>C</u>H_3), 114.67, 115.38, 119.15, 122.02, 125.20, 129.56, 131.80, 134.47, 145.05, 160.55 (C-2), 189.86 (C-3').

HRMS: *m/z* Calculated for $C_{28}H_{21}N_4O_3$ $[M]^+$ 480.1528 found 480.1565.

(*E*)-3-(3-(4-fluorophenyl)-3-oxoprop-1-en-1-yl)-1-((1-(4-methoxyphenyl)-1*H*-1,2,3-triazol-4-yl)methyl)quinolin-2(1*H*)-one (**25**)

Yield: 72%, yellow solid.

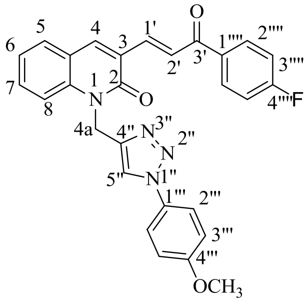

25

Melting Point: 211-213 °C.

IR (KBr) (cm^{-1}): ν 2887 (sp^3 C-H), 1721 (C=O), 1623 (C=C), 1080 (C-O-C), 822 (*p*-substitution).

^{1}H NMR (400 MHz, $CDCl_3$): δ 3.80 (3H, s, -OMe), 5.69 (2H, s, C-4aH), 6.92-6.94 (2H, m, Ar-H), 7.12-7.14 (2H, m, Ar-H), 7.25-7.27 (1H, m, Ar-H), 7.54-7.56 (3H, m, Ar-H), 7.60-7.65 (2H, m, Ar-H), 7.78 (1H, d, *J* = 15.57 Hz, C-2'H), 7.97-8.02 (3H, m,

Ar-H), 8.09-8.13 (1H, m, Ar-H), 8.45 ((1H, d, *J* = 15.57 Hz, C-1'H).

^{13}C NMR (100 MHz, $CDCl_3$): δ 38.68 (C-4a), 55.55 (-O<u>C</u>H_3), 111.68, 114.66, 115.28, 115.56, 120.31, 120.33, 121.86, 122.08, 123.02, 125.34, 125.68, 129.64, 130.26, 131.25, 131.34, 132.48, 139.12, 140.05, 142.68, 143.73, 159.80, 160.90 (C-2), 189.28 (C-3').

HRMS: *m/z* Calculated for $C_{28}H_{21}N_4O_3$ $[M]^+$ 480.1528 found 480.1550.

(*E*)-3-(3-(4-chlorophenyl)-3-oxoprop-1-en-1-yl)-1-((1-(4-methoxyphenyl)-1*H*-1,2,3-triazol-4-yl)methyl)quinolin-2(1*H*)-one (**26**)

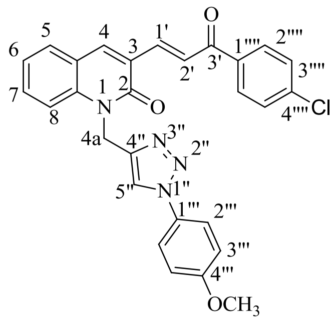

26

Yield: 70%, yellow solid.

Melting Point: 230-232 ^{o}C.

IR (KBr) (cm^{-1}): ν 2867 (sp^3 C-H), 1710 (C=O), 1625 (C=C), 1080 (C-O-C), 818 (*p*-substitution).

^{1}H NMR (400 MHz, $CDCl_3$): δ 3.75 (3H, s, -OMe), 5.64 (2H, s, C-4aH), 6.87-6.89 (2H, m, Ar-H), 7.19-7.23 (1H, m, Ar-H), 7.38-7.41 (2H, m, Ar-H), 7.50-7.61 (4H, m, Ar-H), 7.74 (1H, d, *J* = 15.11 Hz, C-2'H), 7.93-7.98 (5H, m, Ar-H), 8.39 ((1H, d, *J* = 15.11 Hz, C-1'H).

^{13}C NMR (100 MHz, $CDCl_3$): δ 38.68 (C-4a), 55.56 (-O<u>C</u>H_3), 114.68, 115.30, 122.11, 123.06, 125.21, 128.89, 129.68, 130.10, 132.55, 140.32, 142.81, 159.82 (C-2), 190.27 (C-3').

HRMS: *m/z* Calculated for $C_{28}H_{21}N_4O_3$ $[M]^+$ 496.1328 found 496.1375.

(*E*)-3-(3-(4-bromophenyl)-3-oxoprop-1-en-1-yl)-1-((1-(4-methoxyphenyl)-1*H*-1,2,3-triazol-4-yl)methyl)quinolin-2(1*H*)-one (**27**)

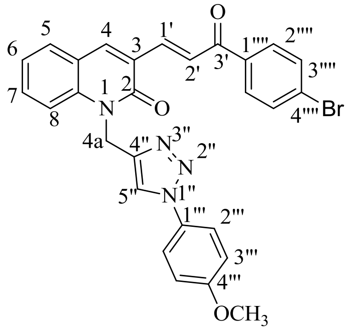

27

Yield: 75%, yellow solid.

Melting Point: 201-203 °C.

IR (KBr) (cm^{-1}): ν 2863 (sp^3 C-H), 1714 (C=O), 1628 (C=C), 1080 (C-O-C), 819 (*p*-substitution).

^{1}H NMR (400 MHz, $CDCl_3$): δ 3.80 (3H, s, -OMe), 5.69 (2H, s, C-4aH), 6.93-6.95 (2H, m, Ar-H), 7.24-7.28 (2H, m, Ar-H), 7.55-7.72 (6H, m, Ar-H), 7.93 (1H, d, *J* = 15.11 Hz, C-2'H), 7.94-8.02 (4H, m, Ar-H), 8.43 ((1H, d, *J* = 15.11 Hz, C-1'H).

^{13}C NMR (100 MHz, $CDCl_3$): δ 38.68 (C-4a), 55.55 (-O<u>C</u>H_3), 114.68, 115.58, 122.11, 125.40, 129.69, 130.23, 131.88, 136.93, 139.17, 140.54, 14261, 159.62 (C-2), 190.09 (C-3').

HRMS: *m/z* Calculated for $C_{28}H_{21}N_4O_3$ $[M]^+$ 540.0797 found 540.0755.

(*E*)-1-((1-(4-methoxyphenyl)-1*H*-1,2,3-triazol-4-yl)methyl)-3-(3-oxo-3-(p-tolyl)prop-1-en-1-yl)quinolin-2(1*H*)-one (**28**)

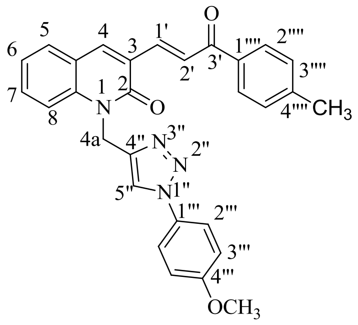

28

Yield: 62%, yellow solid.

Melting Point: 213-215 °C.

IR (KBr) (cm^{-1}): ν 2950 (sp^3 C-H), 1720 (C=O), 1729 (C=C), 1080 (C-O-C), 820 (*p*-substitution).

^{1}H NMR (400 MHz, $CDCl_3$): δ 21.68 (3H, s, -CH_3), 3.81 (3H, s, -OMe), 5.70 (2H, s, C-4aH), 6.93-6.95 (2H, m, Ar-H), 7.24-7.29 (2H, m, Ar-H), 7.54-7.65 (5H, m, Ar-H), 7.79 (1H, d, *J* = 15.57 Hz, C-2'H), 7.97-8.03 (5H, m, Ar-H), 8.45 (1H, m, Ar-H).

^{13}C NMR (100 MHz, $CDCl_3$): δ 38.61 (C-4a), 55.51 (-O<u>C</u>H_3), 55.78 (-O<u>C</u>H_3),

111.68, 114.60, 115.15, 120.31, 120.61, 121.85, 122.00, 122.84, 126.34, 129.20, 129.49, 130.23, 132.07, 132.81, 138.05, 139.14, 140.29, 143.80, 158.14, 159.70, 160.85 (C-2), 193.39 (C-3’).

HRMS: *m/z* Calculated for $C_{29}H_{24}N_4O_3$ $[M]^+$ 476.1828 found 476.1855.

(*E*)-1-((1-(4-methoxyphenyl)-1*H*-1,2,3-triazol-4-yl)methyl)-3-(3-(2-methoxyphenyl)-3-oxoprop-1-en-1-yl)quinolin-2(1*H*)-one (**29**)

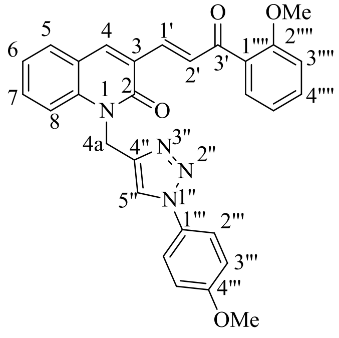

29

Yield: 65%, yellow solid.

Melting Point: 210-212 °C.

IR (KBr) (cm^{-1}): ν 2955 (sp^3 C-H), 1725 (C=O), 1629 (C=C), 1080 (C-O-C), 822 (*p*-substitution).

^{1}H NMR (400 MHz, $CDCl_3$): δ 3.80 (3H, s, -OMe), 3.81 (3H, s, -OMe), 5.66 (2H, s, C-4aH), 6.93 (2H, d, *J* = 8.70 Hz, Ar-H), 7.03-7.05 (1H, m, Ar-H), 7.25-7.27 (2H, m, Ar-H), 7.44-7.45 (1H, m, Ar-H), 7.54 (2H, d, *J* = 8.70 Hz, Ar-H), 7.60-7.63 (3H, m, Ar-H), 7.72 (1H, d, *J* = 15.16 Hz, C-2’H), 7.77-7.79 (1H, m, Ar-H), 7.99 (2H, d, *J* = 7.79, Ar-H).

^{13}C NMR (100 MHz, $CDCl_3$): δ 38.61 (C-4a), 55.51 (-O<u>C</u>H_3), 55.78 (-O<u>C</u>H_3), 111.68, 114.60, 115.15, 120.31, 120.61, 121.85, 122.00, 122.84, 126.34, 129.20, 129.49, 130.23, 132.07, 132.81, 138.05, 139.14, 140.29, 143.80, 158.14, 159.70, 160.85 (C-2), 193.39 (C-3’).

HRMS: *m/z* Calculated for $C_{29}H_{24}N_4O_4$ $[M]^+$ 492.1828 found 492.1855.

(*E*)-1-((1-(4-methoxyphenyl)-1*H*-1,2,3-triazol-4-yl)methyl)-3-(3-(3-methoxyphenyl)-3-oxoprop-1-en-1-yl)quinolin-2(1*H*)-one (30)

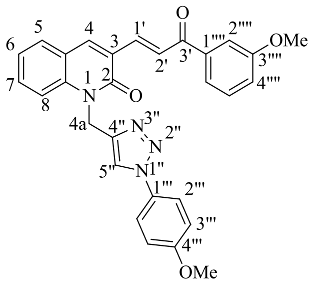

30

Yield: 55%, light yellow solid.

Melting Point: 196-198 °C.

IR (KBr) (cm^{-1}): ν 2989 (sp^3 C-H), 1695 (C=O), 1590 (C=C), 1100 (C-O-C), 822 (*p*-substitution).

^{1}H NMR (400 MHz, $CDCl_3$): δ 3.79 (3H, s, -OMe), 3.85 (3H, s, -OMe), 5.69 (2H, s, C-4aH), 6.92 (2H, d, *J* = 9.16 Hz, Ar-H), 7.11-7.12 (1H, m, Ar-H), 7.24-7.25 (1H, m, Ar-H), 7.40-7.42 (2H, m, Ar-H), 7.54-7.63 (4H, m , Ar-H), 7.80 (1H, d, *J* = 15.57 Hz, C-2'H), 7.95-7.98 (2H, m, Ar-H), 8.01 (1H, s, C-5''H), 8.39 (1H, d, *J* = 15.57 Hz, C-1'H).

^{13}C NMR (100 MHz, $CDCl_3$): δ 38.68 (C-4a), 55.46 (-O$\underline{C}H_3$), 55.54 (-O$\underline{C}H_3$), 112.76, 114.62, 115.22, 119.42, 120.31, 121.39, 121.80, 122.05, 122.94, 125.74, 125.84, 129.50, 129.59, 130.26, 132.33, 139.11, 139.41, 139.78, 142.15, 159.76, 159.81, 160.86 (C-2), 163.49, 189.20 (C-3').

HRMS: *m/z* Calculated for $C_{29}H_{24}N_4O_4$ $[M]^+$ 492.1828 found 492.1790.

(*E*)-1-((1-(4-methoxyphenyl)-1*H*-1,2,3-triazol-4-yl)methyl)-3-(3-(4-methoxyphenyl)-3-oxoprop-1-en-1-yl)quinolin-2(1*H*)-one (31)

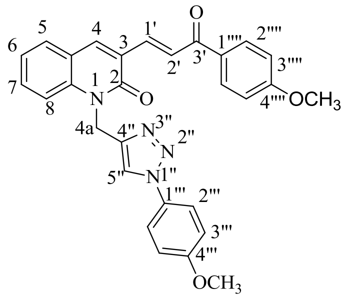

31

Yield: 65%, light yellow solid.

Melting Point: 190-192°C.

IR (KBr) (cm^{-1}): ν 2982 (sp^3 C-H), 1705 (C=O), 1593 (C=C), 1103 (C-O-C), 820 (*p*-substitution).

^{1}H NMR (400 MHz, $CDCl_3$): δ 3.79 (3H, s, -OMe), 3.85 (3H, s, -OMe), 5.69 (2H, s, C-4aH), 6.91-6.95 (4H, m, Ar-H), 7.24-7.25 (1H, m, Ar-H), 7.54-7.63 (4H, m,

Ar-H), 7.76 (1H, d, *J* = 15.57 Hz, C-2'H), 7.95-8.09 (5H, m, Ar-H), 8.45 (1H, d, *J* = 15.57 Hz, C-1'H).

^{13}C NMR (100 MHz, $CDCl_3$): δ 38.68 (C-4a), 55.46 (-O<u>C</u>H_3), 55.54 (-O<u>C</u>H_3), 113.76, 114.64, 115.21, 120.39, 121.86, 122.08, 122.94, 125.76, 125.98, 129.55, 130.27, 131.03, 132.24, 139.03, 142.14, 143.81, 159.77, 163.45 (C-2), 189.20 (C-3').

HRMS: *m/z* Calculated for $C_{29}H_{24}N_4O_4$ $[M]^+$ 492.1728 found 492.1790.

(*E*)-3-(3-(2-fluorophenyl)-3-oxoprop-1-en-1-yl)-1-((1-(*p*-tolyl)-1*H*-1,2,3-triazol-4-yl)methyl)quinolin-2(1*H*)-one (32)

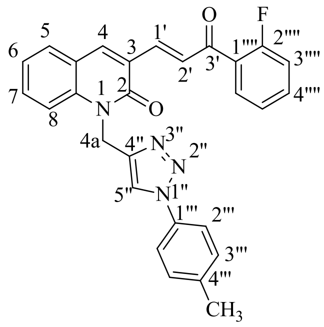

32

Yield: 88%, yellow solid.

Melting Point: 197-199 °C.

IR (KBr) (cm^{-1}): ν 2955 (sp^3 C-H), 1705 (C=O), 1590 (C=C), 1080 (C-F), 822 (*p*-substitution).

^{1}H NMR (400 MHz, $CDCl_3$): δ 2.35 (3H, s, -CH_3), 5.67 (2H, s, C-4aH), 7.17-7.20, (1H, m, Ar-H), 7.22-7.25 (4H, m, Ar-H), 7.52-7.54 (3H, m, Ar-H), 7.60-7.62 (2H, m, Ar-H), 7.78-7.82 (2H, m, Ar-H), 8.00-8.03 (2H, m, Ar-H), 8.07-8.12 (2H, m, Ar-H).

^{13}C NMR (100 MHz, $CDCl_3$): δ 21.01 (-CH_3), 38.62 (C-4a), 115.25, 116.66, 120.30, 122.93, 124.41, 125.83, 129.64, 130.10, 130.86, 139.60, 141.44, 160.94 (C-2), 189.67 (C-3').

HRMS: *m/z* Calculated for $C_{28}H_{21}FN_4O_2$ $[M]^+$ 464.1624 found 464.1615.

(*E*)-3-(3-(3-fluorophenyl)-3-oxoprop-1-en-1-yl)-1-((1-(p-tolyl)-1*H*-1,2,3-triazol-4-yl)methyl)quinolin-2(1*H*)-one (33)

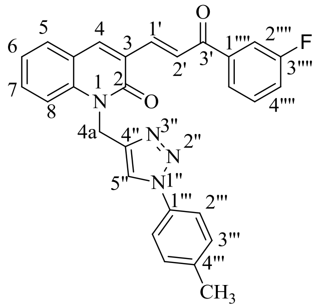

33

Yield: 78%, yellow solid.

Melting Point: 210-212 °C.

IR (KBr) (cm^{-1}): ν 2950 (sp^3 C-H), 1700 (C=O), 1580 (C=C), 1080 (C-F), 822 (*p*-substitution).

^{1}H NMR (400 MHz, $CDCl_3$): δ 2.34 (3H, s, -CH_3), 5.69 (2H, s, C-4aH), 7.21-7.28, (4H, m, Ar-H), 7.42-7.54 (3H, m, Ar-H), 7.60-7.65 (2H, m, Ar-H), 7.68-7.87 (3H, m, Ar-H), 7.97-8.08 (3H, m, Ar-H), 8.41 (1H, d, *J* = 15.11 Hz, Ar-H).

^{13}C NMR (100 MHz, $CDCl_3$): δ 21.02 (-CH_3), 38.65 (C-4a), 115.27, 119.72, 119.93, 120.33, 123.10, 124.37, 125.18, 125.58, 129.67, 130.13, 132.53, 138.92, 140.51, 142.80, 161.31 (C-2), 190.05 (C-3').

HRMS: *m/z* Calculated for $C_{28}H_{21}FN_4O_2$ $[M]^+$ 464.1624 found 464.1615.

(*E*)-3-(3-(4-fluorophenyl)-3-oxoprop-1-en-1-yl)-1-((1-(p-tolyl)-1*H*-1,2,3-triazol-4-yl)methyl)quinolin-2(1*H*)-one (34)

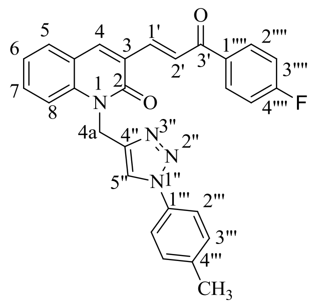

34

Yield: 70%, yellow solid.

Melting Point: 215-217 °C.

IR (KBr) (cm^{-1}): ν 2940 (sp^3 C-H), 1710 (C=O), 1600 (C=C), 1070 (C-F), 820 (*p*-substitution).

^{1}H NMR (400 MHz, $CDCl_3$): δ 2.34 (3H, s, -CH_3), 5.69 (2H, s, C-4aH), 7.11-7.26, (5H, m, Ar-H), 7.52-7.54 (2H, m, Ar-H), 7.59-7.64 (2H, m, Ar-H), 7.77 (1H, d, *J* = 15.11 Hz, C-2'H), 7.96-7.98 (2H, m, Ar-H), 8.08-8.12 (3H, m, Ar-H), 8.45 (1H, d, *J* = 15.11 Hz, C-1'H).

^{13}C NMR (100 MHz, $CDCl_3$): δ 21.02 (-CH_3), 38.65 (C-4a), 115.24, 115.53, 115.75,

120.32, 122.99, 125.30, 125.65, 129.63, 130.12, 131.22, 131.31, 132.44, 138.92, 139.08, 140.01, 142.66, 162.81 (C-2), 190.04 (C-3').

HRMS: *m/z* Calculated for $C_{28}H_{21}FN_4O_2$ $[M]^+$ 464.1636 found 464.1615.

(*E*)-3-(3-(4-chlorophenyl)-3-oxoprop-1-en-1-yl)-1-((1-(p-tolyl)-1*H*-1,2,3-triazol-4-yl)methyl)quinolin-2(1*H*)-one (35)

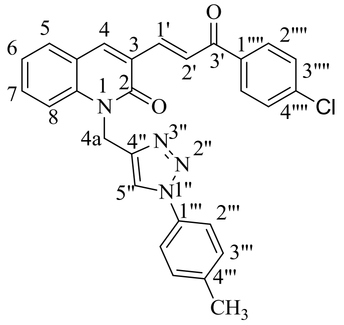

35

Yield: 80%, yellow solid.

Melting Point: 196-198 °C.

IR (KBr) (cm^{-1}): ν 2950 (sp^3 C-H), 1720 (C=O), 1605 (C=C), 1080 (C-F), 822 (*p*-substitution).

^{1}H NMR (400 MHz, $CDCl_3$): δ 2.35 (3H, s, $-CH_3$), 5.69 (2H, s, C-4aH), 7.24-7.27, (4H, m, Ar-H), 7.38-7.54 (4H, m, Ar-H), 7.61-7.72 (2H, m, Ar-H), 7.79 (1H, d, *J* = 15.11 Hz, C-2'H), 8.00-8.03 (3H, m, Ar-H), 8.44 (1H, d, *J* = 15.11 Hz, C-1'H).

^{13}C NMR (100 MHz, $CDCl_3$): δ 21.07 ($-CH_3$), 38.80 (C-4a), 115.30, 120.39, 123.07, 125.22, 128.89, 129.69, 130.11, 131.18, 132.56, 140.35, 142.86, 161.51 (C-2), 189.49 (C-3').

HRMS: *m/z* Calculated for $C_{28}H_{21}ClN_4O_2$ $[M]^+$ 480.1353 found 480.1315.

(*E*)-3-(3-(4-bromophenyl)-3-oxoprop-1-en-1-yl)-1-((1-(p-tolyl)-1*H*-1,2,3-triazol-4-yl)methyl)quinolin-2(1*H*)-one (36)

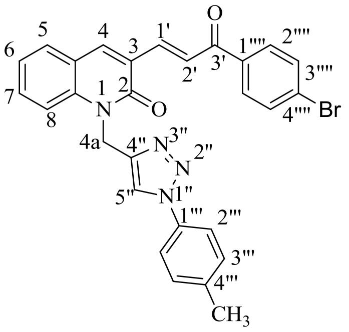

36

Yield: 85%, yellow solid.

Melting Point: 216-218 °C.

IR (KBr) (cm^{-1}): ν 2850 (sp^3 C-H), 1740 (C=O), 1615 (C=C), 1070 (C-F).

^{1}H NMR (400 MHz, $CDCl_3$): δ 2.40 (3H, s, $-CH_3$), 5.69 (2H, s, C-4aH), 7.21-7.27, (6H, m, Ar-H), 7.52-7.54 (3H, m, Ar-H), 7.59-7.63 (2H, m, Ar-H), 7.78 (1H, d, *J* =

15.57 Hz, C-2'H), 7.95-8.00 (2H, m, Ar-H),8.08 (1H, s, C-5''), 8.44 (1H, d, *J* = 15.57 Hz, C-1'H).

^{13}C NMR (100 MHz, $CDCl_3$): δ 21.01 (-CH_3), 38.63 (C-4a), 115.17, 120.29, 122.92, 125.75, 125.88, 128.80, 129.24, 129.56, 130.10, 131.18, 132.26, 138.86, 139.36, 142.17, 143.69, 160.72 (C-2), 190.36 (C-3').

HRMS: *m/z* Calculated for $C_{28}H_{21}BrN_4O_2$ $[M]^+$ 524.0848 found 524.0890.

(*E*)-3-(3-oxo-3-(*p*-tolyl)prop-1-en-1-yl)-1-((1-(*p*-tolyl)-1*H*-1,2,3-triazol-4-yl)methyl)quinolin-2(1*H*)-one (**37**)

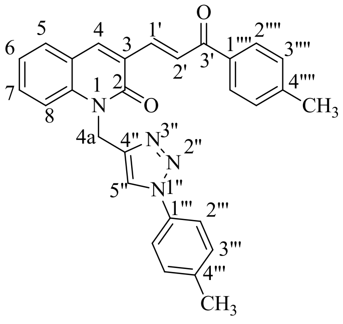

37

Yield: 92%, yellow solid.

Melting Point: 202-204 °C.

IR (KBr) (cm^{-1}): ν 3020 (sp^3 C-H), 1685 (C=O), 1609 (C=C), 832 (*p*-substitution).

^{1}H NMR (400 MHz, $CDCl_3$): δ 2.14 (3H, s, -CH_3), 2.35 (3H, s, -CH_3), 5.69 (2H, s, C-4aH), 7.52-7.62 (8H, m, Ar-H), 7.79 (1H, d, *J* = 15.11 Hz, C-2'H), 7.95-8.00 (4H, m, Ar-H), 8.07 (1H, s, C-5''H), 8.43 (1H, d, *J* = 15.57 Hz, C-1'H).

^{13}C NMR (100 MHz, $CDCl_3$): δ 21.06 (-CH_3), 38.69 (C-4a), 115.17, 120.29, 122.92, 125.75, 125.88, 128.80, 129.24, 129.56, 130.10, 132.26, 138.86, 139.38, 142.17, 143.69, 160.96 (C-2), 189.84 (C-3').

HRMS: *m/z* Calculated for $C_{29}H_{24}N_4O_2$ $[M]^+$ 460.1976 found 460.1951.

(*E*)-3-(3-(2-methoxyphenyl)-3-oxoprop-1-en-1-yl)-1-((1-(*p*-tolyl)-1*H*-1,2,3-triazol-4-yl)methyl)quinolin-2(1*H*)-one (38)

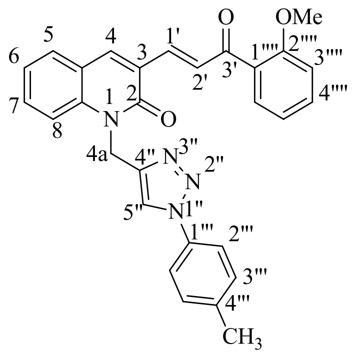

38

Yield: 90%, yellow solid.

Melting Point: 153-155 °C.

IR (KBr) (cm^{-1}): ν 3035, (sp^3 C-H), 1665 (C=O), 1620 (C=C), 1090 (C-O-C), 822 (*p*-substitution).

^{1}H NMR (400 MHz, $CDCl_3$): δ 2.36 (3H, s, $-CH_3$), 3.89 (3H, s, $-OCH_3$), 5.66 (2H, s, C-4aH), 6.98-7.03 (2H, m, Ar-H), 7.21-7.25 (4H, m , Ar-H), 7.45-7.47 (1H, m, Ar-H), 7.51-7.53 (2H, m, Ar-H), 7.60-7.62 (3H, m, Ar-H), 7.72 (1H, d, *J* = 15.57 Hz, C-2'H), 7.94-7.98 (3H, m, Ar-H), 8.04 (1H, s, C-5''H).

^{13}C NMR (100 MHz, $CDCl_3$): δ 21.02 ($-CH_3$), 38.61 (C-4a), 55.78 ($-OCH_3$), 111.63, 115.15, 120.28, 120.61, 122.87, 129.50, 130.11, 130.25, 132.09, 132.83, 138.08, 139.13, 140.33, 158.63, 161.45 (C-2), 193.80 (C-3').

HRMS: *m/z* Calculated for $C_{29}H_{24}N_4O_3$ $[M]^+$ 476.1854 found 476.1833.

(*E*)-3-(3-(3-methoxyphenyl)-3-oxoprop-1-en-1-yl)-1-((1-(*p*-tolyl)-1*H*-1,2,3-triazol-4-yl)methyl)quinolin-2(1*H*)-one (39)

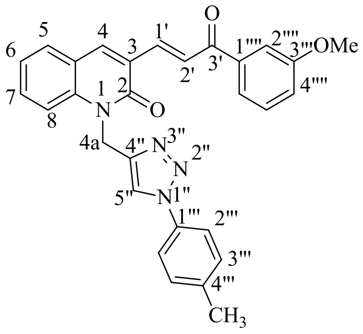

39

Yield: 91%, yellow solid.

Melting Point: 192-194 °C.

IR (KBr) (cm^{-1}): ν 2956 (sp^3 C-H), 1695 (C=O), 1588 (C=C), 1099 (C-O-C), 822 (*p*-substitution).

^{1}H NMR (400 MHz, $CDCl_3$): δ 2.34 (3H, s, $-CH_3$), 3.85 (3H, s, $-OCH_3$), 5.69 (2H, s, C-4aH), 7.11-7.15 (1H, m, Ar-H), 7.25-7.27 (1H, m, Ar-H), 7.53-7.61 (5H, m, Ar-H), 7.68 (1H, d, *J* = 7.33 Hz, Ar-H), 7.80 (1H, d, *J* = 15.57 Hz, C-2'H), 7.82-7.99 (2H, m, Ar-H), 8.09 (1H, s, C-5''H), 8.41 (1H, d, *J* = 15.57 Hz, C-1'H).

^{13}C NMR (100 MHz, $CDCl_3$): δ 21.02 (-CH_3), 38.21 (C-4a), 55.42 (-OCH_3), 112.70, 115.19, 119.43, 120.29, 121.38, 122.93, 125.70, 129.49, 129.59, 130.11, 132.33, 138.87, 139.79, 142.20, 159.78 (C-2), 160.69, 189.82 (C-3').

HRMS: *m/z* Calculated for $C_{29}H_{24}N_4O_3$ $[M]^+$ 476.1854 found 476.1866.

(*E*)-3-(3-(4-methoxyphenyl)-3-oxoprop-1-en-1-yl)-1-((1-(p-tolyl)-1*H*-1,2,3-triazol-4-yl)methyl)quinolin-2(1*H*)-one (40)

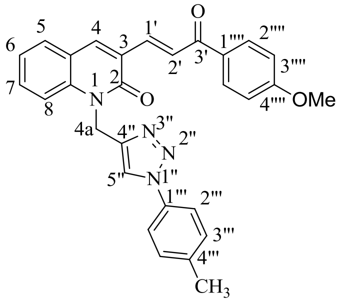

40

Yield: 67%, yellow solid.

Melting Point: 182-184 °C.

IR (KBr) (cm^{-1}): ν 2946 (sp^3 C-H), 1675 (C=O), 1580 (C=C), 1090 (C-O-C), 820 (*p*-substitution).

^{1}H NMR (400 MHz, $CDCl_3$): δ 2.34 (3H, s, -CH_3), 3.85 (3H, s, -OCH_3), 5.69 (2H, s, C-4aH), 6.93-6.95 (2H, m, Ar-H), 7.21-7.25 (3H, m, Ar-H), 7.52-7.54 (4H, m, Ar-H), 7.76 (1H, d, *J* = 15.57 Hz, C-2'H), 7.95-7.97 (2H, m, Ar-H), 8.07-8.10 (1H, m, Ar-H), 8.47 (1H, d, *J* = 15.11 Hz, C-1'H).

^{13}C NMR (100 MHz, $CDCl_3$): δ 21.03 (-CH_3), 38.67 (C-4a), 55.45 (-OCH_3), 113.74, 115.17, 120.34, 122.92, 125.72, 125.95, 129.55, 130.13, 132.22, 138.89, 139.02, 142.16, 160.93 (C-2), 163.43, 189.15 (C-3').

HRMS: *m/z* Calculated for $C_{29}H_{24}N_4O_3$ $[M]^+$ 476.1854 found 476.1866.

(*E*)-1-((1-(4-fluorophenyl)-1*H*-1,2,3-triazol-4-yl)methyl)-3-(3-(2-fluorophenyl)-3-oxoprop-1-en-1-yl)quinolin-2(1*H*)-one (41)

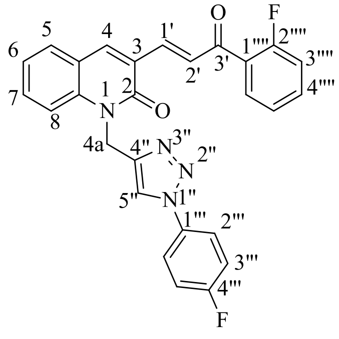

41

Yield: 82%, yellow solid.

Melting Point: 186-188 ^{o}C.

IR (KBr) (cm^{-1}): ν 2955 (sp^3 C-H), 1675 (C=O), 1592 (C=C), 1028 (C-F), 818 (*p*-substitution).

^{1}H NMR (400 MHz, $CDCl_3$): δ 5.68 (2H, s, C-4aH), 7.12-7.18 (3H, m, Ar-H), 7.21-7.28 (3H, m, Ar-H), 7.50-7.52 (1H, m, Ar-H), 7.57-7.66 (4H, m, Ar-H), 7.79-7.83 (2H, m, Ar-H), 7.97 (1H, d, *J* = 8.7 Hz, Ar-H), 8.02 (1H, s, C-5’’H), 8.06-8.08 (2H, m, Ar-H).

^{13}C NMR (100 MHz, $CDCl_3$): δ 38.54 (C-4a), 115.13, 117.13, 123.03, 125.56, 129.71, 129.82, 130.91, 132.44, 139.51, 141.52, 160.92 (C-2), 186.64 (C-3’).

HRMS: *m/z* Calculated for $C_{27}H_{18}F_2N_4O_2$ $[M]^+$ 468.1434 found 468.1435.

(*E*)-1-((1-(4-fluorophenyl)-1*H*-1,2,3-triazol-4-yl)methyl)-3-(3-(3-fluorophenyl)-3-oxoprop-1-en-1-yl)quinolin-2(1*H*)-one (42)

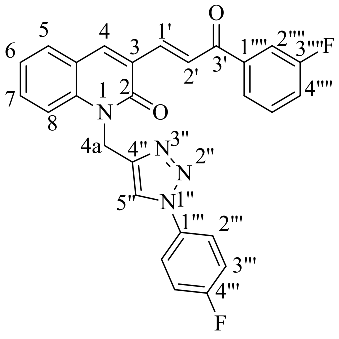

42

Yield: 68%, yellow solid.

Melting Point: 203-205 ^{o}C.

IR (KBr) (cm^{-1}): ν 3030 (sp^3 C-H), 1665 (C=O), 1590 (C=C), 1020 (C-F), 820 (*p*-substitution).

^{1}H NMR (400 MHz, $CDCl_3$): δ 5.68 (2H, s, C-4aH), 7.11-7.15 (3H, m, Ar-H),7.25-7.27 (3H, m, Ar-H), 7.47-7.49 (1H, m, Ar-H), 7.63-7.65 (2H, m, Ar-H), 7.71-7.74 (2H, m, Ar-H), 7.79 (1H, d, *J* = 15.11 Hz, C-2’H), 7.84-7.86 (1H, m, Ar-H), 7.95-7.99 (3H, m, Ar-H), 8.09 (1H, s, C-5’’H), 8.39 (1H, d, *J* = 15.57 Hz, C-1’H).

^{13}C NMR (100 MHz, $CDCl_3$): δ 38.57 (C-4a), 115.17, 116.52, 116.74, 119.97,

120.28, 121.99, 122.39, 122.47, 123.07, 124.35, 125.55, 129.72, 130.17, 130.25, 132.56, 139.09, 140.08, 140.14, 140.46, 142.82, 144.04, 160.84, 161.13, 161.59, 163.61 (C-2), 189.51 (C-3').

HRMS: *m/z* Calculated for $C_{27}H_{18}F_2N_4O_2$ $[M]^+$ 468.1434 found 468.1431.

(*E*)-1-((1-(4-fluorophenyl)-1*H*-1,2,3-triazol-4-yl)methyl)-3-(3-(4-fluorophenyl)-3-oxoprop-1-en-1-yl)quinolin-2(1*H*)-one (43)

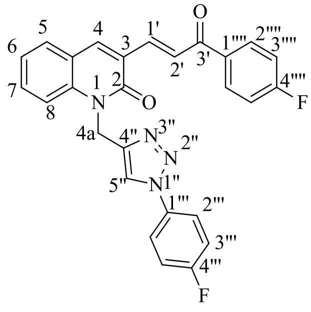

43

Yield: 87%, yellow solid.

Melting Point: 202-204 °C.

IR (KBr) (cm^{-1}): ν 2990 (sp^3 C-H), 1665 (C=O), 1610 (C=C), 1080 (C-F), 810 (*p*-substitution).

^{1}H NMR (400 MHz, $CDCl_3$): δ 5.70 (2H, s, C-4aH), 7.21-7.25 (3H, m, Ar-H), 7.38-7.44 (4H, m, Ar-H), 7.64-7.67 (3H, m, Ar-H), 7.84-7.87 (3H, m, Ar-H), 7.88-8.00 (2H, m, Ar-H), 8.11 (1H, s, C-5''H), 8.42 (1H, d, *J* = 15.11 Hz, C-1'H).

^{13}C NMR (100 MHz, $CDCl_3$): δ 37.95 (C-4a), 115.22, 120.47, 123.04, 125.15, 128.81, 129.65, 132.56, 139.08, 140.49, 142.87, 161.43, 164.02 (C-2), 189.42 (C-3').

HRMS: *m/z* Calculated for $C_{27}H_{18}F_2N_4O_2$ $[M]^+$ 468.1434 found 468.1421.

(*E*)-3-(3-(4-chlorophenyl)-3-oxoprop-1-en-1-yl)-1-((1-(4-fluorophenyl)-1*H*-1,2,3-triazol-4-yl)methyl)quinolin-2(1*H*)-one (44)

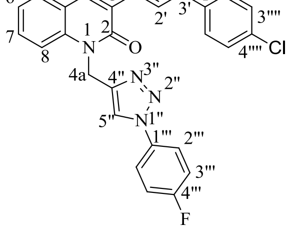

44

Yield: 88%, yellow solid.

Melting Point: 190-192 °C.

IR (KBr) (cm^{-1}): ν 2890 (sp^3 C-H), 1660 (C=O), 1620 (C=C), 1080 (C-F), 820 (*p*-substitution).

^{1}H NMR (400 MHz, $CDCl_3$): δ 5.69 (2H, s, C-4aH), 7.12-7.16 (3H, m, Ar-H), 7.24-7.30 (2H, m, Ar-H), 7.41-7.47 (2H, m, Ar-H), 7.61-7.66 (3H, m, Ar-H), 7.75-7.80 (1H, m, Ar-H), 7.96-8.02 (3H, m, Ar-H), 8.08 (1H, s, C-5''H), 8.43 (1H, d, *J* = 15.11 Hz, C-1'H).

^{13}C NMR (100 MHz, $CDCl_3$): δ 38.58 (C-4a), 115.18, 116.54, 116.77, 120.31, 121.99, 122.41, 122.49, 125.18, 125.59, 128.88, 129.72, 130.07, 132.56, 136.29, 139.07, 139.30, 140.26, 142.82, 144.05, 160.88 (C-2), 189.56 (C-3').

HRMS: *m/z* Calculated for $C_{27}H_{18Cl}FN_4O_2$ $[M]^+$ 484.1434 found 468.1421

(*E*)-3-(3-(4-bromophenyl)-3-oxoprop-1-en-1-yl)-1-((1-(4-fluorophenyl)-1*H*-1,2,3-triazol-4-yl)methyl)quinolin-2(1*H*)-one (**45**)

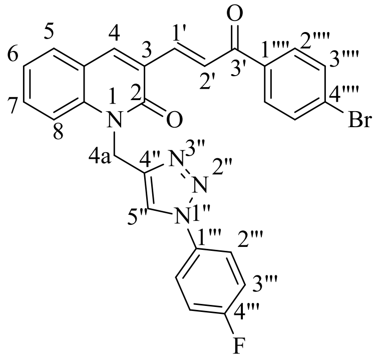

45

Yield: 80%, yellow solid.

Melting Point: 238-240 °C.

IR (KBr) (cm^{-1}): ν 2995 (sp^3 C-H), 1700 (C=O), 1590 (C=C), 1098 (C-F), 820 (*p*-substitution) 522 (C-Br).

^{1}H NMR (400 MHz, $CDCl_3$): δ 5.66 (2H, s, C-4aH), 6.84 (1H, d, *J* = 12.97, Ar-H), 7.08-7.13 (3H, m, Ar-H), 7.21-7.25 (1H, m , Ar-H), 7.49 (1H, d, *J* = 8.39 Hz, Ar-H), 7.54-7.62 (3H, m, Ar-H), 7.75 (1H, d, *J* = 15.26 Hz, C-2'H), 7.79-7.81 (1H, m, Ar-H), 7.90-7.97 (4H, m, Ar-H), 8.02 (1H, s, C-4H), 8.11 (1H, s, C-5''H), 8.38 (1H, d, *J* = 16.02 Hz, C-1').

^{13}C NMR (100 MHz, $CDCl_3$): δ 38.58 (C-4a), 115.18, 116.54, 116.77, 120.31, 121.99, 122.41, 122.49, 123.09, 125.18, 125.59, 128.88, 129.72, 130.07, 132.56, 136.29, 139.07, 139.50, 140.26, 142.82 51, 144.05, 160.88 (C-2), 163.21, 189.56 (C-3').

HRMS: *m/z* Calculated for $C_{27}H_{18}BrFN_4O_2$ $[M]^+$ 528.0645 found 528.0631.

(*E*)-1-((1-(4-fluorophenyl)-1*H*-1,2,3-triazol-4-yl)methyl)-3-(3-oxo-3-(p-tolyl)prop-1-en-1-yl)quinolin-2(1*H*)-one (**46**)

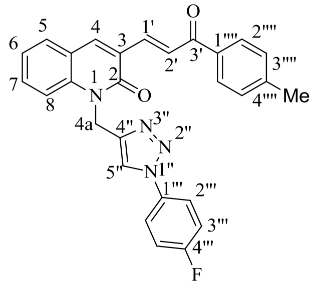

46

Melting Point: 132-134 °C.

IR (KBr) (cm^{-1}): ν 2985 (sp^3 C-H), 1670 (C=O), 1620 (C=C), 1020 (C-O-C), 820 (*p*-substitution).

^{1}H NMR (400 MHz, $CDCl_3$): δ 2.41 (3H, s, -CH_3), 5.70 (2H, s, C-4aH), 7.12-7.16 (3H, m, Ar-H), 7.24-7.28 (3H, m, Ar-H), 7.58-7.67 (4H, m, Ar-H), 7.71 (1H, d, *J* = 15.57 Hz, C-2'H), 7.95-7.99 (3H, m, Ar-H), 8.09 (1H, s, C-5''H), 8.44 (1H, d, *J* = 15.57 Hz, C-1').

^{13}C NMR (100 MHz, $CDCl_3$): δ 21.67 (-$\underline{C}H_3$), 38.52 (C-4a), 115.09, 116.50, 116.73, 120.34, 121.96, 122.38, 122.47, 122.98, 125.77, 125.88, 128.80, 129.26, 129.62, 132.30, 133.04, 138.97, 139.30, 142.17, 143.75, 144.13, 160.91 (C-2), 190.31 (C-3').

HRMS: *m/z* Calculated for $C_{28}H_{21}FN_4O_2$ $[M]^+$ 464.1634 found 464.1613.

(*E*)-1-((1-(4-fluorophenyl)-1*H*-1,2,3-triazol-4-yl)methyl)-3-(3-(2-methoxyphenyl)-3-oxoprop-1-en-1-yl)quinolin-2(1*H*)-one (**47**)

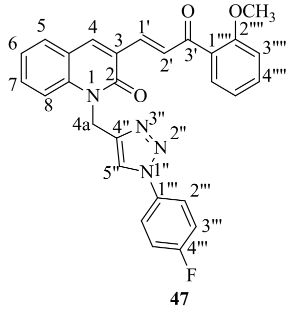

47

Yield: 55%, light brown solid.

Melting Point: 132-134 °C.

IR (KBr) (cm^{-1}): ν 2995 (sp^3 C-H), 1680 (C=O), 1610 (C=C), 1010 (C-O-C), 822 (*p*-substitution).

^{1}H NMR (400 MHz, $CDCl_3$): δ 3.88 (3H, s, –OCH_3), 5.65 (2H, s, C-4aH), 6.98-7.02 (3H, m, Ar-H), 7.13-7.15 (3H, m, Ar-H), 7.27-7.29 (2H, m, Ar-H), 7.45-7.47 (1H, m, Ar-H), 7.60-7.64 (3H, m, Ar-H), 7.71 (1H, d, *J* = 16.03 Hz, C-2'H), 7.97 (3H, m, Ar-H), 8.05 (1H, s, C-5''H).

^{13}C NMR (100 MHz, $CDCl_3$): δ 38.52 (C-4a), 55.78 (-OC̲H₃), 111.63, 115.05, 116.49, 116.72, 120.31, 120.61, 122.34, 122.43, 122.90, 126.31, 129.41, 129.55, 130.24, 132.10, 132.86, 137.98, 139.07, 140.33, 158.12, 160.86 (C-2), 193.36 (C-3').

HRMS: *m/z* Calculated for $C_{28}H_{21}FN_4O_3$ $[M]^+$ 480.1634 found 480.1613.

(*E*)-1-((1-(4-fluorophenyl)-1*H*-1,2,3-triazol-4-yl)methyl)-3-(3-(3-methoxyphenyl)-3-oxoprop-1-en-1-yl)quinolin-2(1*H*)-one (48)

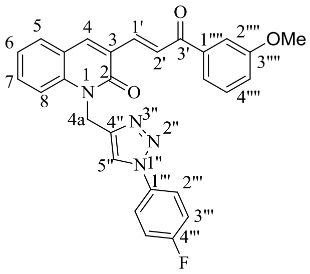

48

Yield: 88%, yellow solid.

Melting Point: 220-222 °C.

IR (KBr) (cm^{-1}): ν 2950 (sp^3 C-H), 1692 (C=O), 1590 (C=C), 1099 (C-O-C), 822 (*p*-substitution).

^{1}H NMR (400 MHz, $CDCl_3$): δ 3.86 (3H, s, $-OCH_3$), 5.70 (2H, s, C-4aH), 6.95 (2H, d, *J* = 9.16 Hz, Ar-H), 7.14-7.17 (3H, m, Ar-H), 7.25-7.26 (1H, m, Ar-H), 7.61-7.65 (3H, m, Ar-H), 7.77 (1H, d, *J* = 15.11 Hz, C-2'H), 7.95-7.98 (2H, m, Ar-H), 8.08-8.10 (3H, m, Ar-H), 8.45 (1H, d, *J* = 15.11 Hz, C-1'H).

^{13}C NMR (100 MHz, $CDCl_3$): δ 38.58 (C-4a), 55.46 (-OC̲H₃), 113.75, 115.09, 116.51, 116.74, 120.38, 121.99, 122.39, 122.47, 122.98, 125.71, 125.93, 129.60, 131.00, 132.25, 138.95, 142.16, 160.95, 163.45 (C-2), 189.10 (C-3').

HRMS: *m/z* Calculated for $C_{28}H_{21}FN_4O_3$ $[M]^+$ 480.1623 found 480.1633.

(*E*)-1-((1-(4-fluorophenyl)-1*H*-1,2,3-triazol-4-yl)methyl)-3-(3-(4-methoxyphenyl)-3-oxoprop-1-en-1-yl)quinolin-2(1*H*) (**49**)

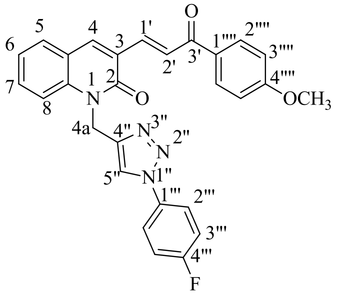

49

Yield: 78%, yellow solid.

Melting Point: 222-224 °C.

IR (KBr) (cm^{-1}): ν 2955 (sp^3 C-H), 1695 (C=O), 1595 (C=C), 1098 (C-O-C), 822 (*p*-substitution).

^{1}H NMR (400 MHz, $CDCl_3$): δ 3.86 (3H, s, $-OCH_3$), 5.70 (2H, s, C-4aH), 6.95 (2H, d, *J* = 9.16 Hz, Ar-H), 7.14-7.17 (3H, m, Ar-H), 7.25-7.26 (1H, m, Ar-H), 7.61-7.65 (3H, m, Ar-H), 7.77 (1H, d, *J* = 15.11 Hz, C-2'H), 7.95-7.98 (2H, m, Ar-H), 8.08-8.10 (3H, m, Ar-H), 8.45 (1H, d, *J* = 15.11 Hz, C-1'H).

^{13}C NMR (100 MHz, $CDCl_3$): δ 38.58 (C-4a), 55.46 ($-O\underline{C}H_3$), 113.75, 115.09, 116.51, 116.74, 120.38, 121.99, 122.39, 122.47, 122.98, 125.71, 125.93, 129.60, 131.00, 132.25, 138.95, 142.16, 160.95, 163.45 (C-2), 189.10 (C-3').

HRMS: *m/z* Calculated for $C_{28}H_{21}FN_4O_3$ $[M]^+$ 480.1623 found 480.1633.

(*E*)-1-((1-(4-chlorophenyl)-1*H*-1,2,3-triazol-4-yl)methyl)-3-(3-(2-fluorophenyl)-3-oxoprop-1-en-1-yl)quinolin-2(1*H*)-one (**50**)

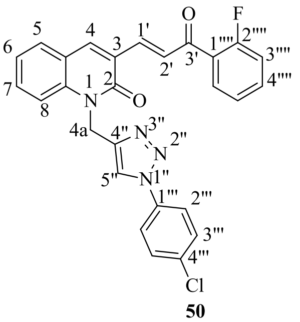

50

Yield: 66%, yellow solid.

Melting Point: 242-244 °C.

IR (KBr) (cm^{-1}): ν 2986 (sp^3 C-H), 1690 (C=O), 1590 (C=C), 1090 (C-F), 820 (*p*-substitution).

^{1}H NMR (400 MHz, $CDCl_3$): δ 5.67 (2H, s, C-4aH), 7.18-7.20 (1H, m, Ar-H), 7.25-7.27 (2H, m, Ar-H), 7.41-7.43 (2H, m, Ar-H), 7.53-7.55 (1H, m, Ar-H), 7.61-7.64 (4H, m, Ar-H), 7.80-7.82 (2H, m, Ar-H), 7.97 (1H, d, *J* = 8.7 Hz, Ar-H), 8.02 (1H, s, C-5''H), 8.10-8.12 (2H, m, Ar-H).

^{13}C NMR (100 MHz, $CDCl_3$): δ 38.54 (C-4a), 115.13, 117.13, 125.56, 123.03, 129.71, 129.82, 130.91, 132.44, 139.51, 141.52, 160.92 (C-2), 189.64 (C-3’).

HRMS: *m/z* Calculated for $C_{27}H_{18}FClN_4O_2$ $[M]^+$ 484.1123 found 484.1123.

(*E*)-1-((1-(4-chlorophenyl)-1*H*-1,2,3-triazol-4-yl)methyl)-3-(3-(3-fluorophenyl)-3-oxoprop-1-en-1-yl)quinolin-2(1*H*)-one (51)

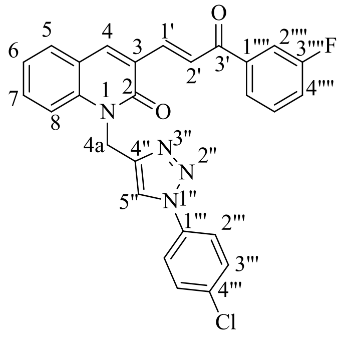

51

Yield: 90%, yellow solid.

Melting Point: 190-192 °C.

IR (KBr) (cm^{-1}): ν 2955 (sp^3 C-H), 1675 (C=O), 1610 (C=C), 825 (*p*-substitution), 820 (C-Cl).

^{1}H NMR (400 MHz, $CDCl_3$): δ 5.70 (2H, s, C-4aH), 7.25-7.29 (2H, m, Ar-H), 7.42-7.49 (3H, m, Ar-H), 7.62-7.68 (4H, m, Ar-H), 7.74-7.76 (1H, m, Ar-H), 7.81 (1H, d, *J* = 15.26 Hz, C-1’H), 7.87 (2H, d, *J* = 7.63 Hz, Ar-H), 7.99-8.02 (2H, m, Ar-H), 8.11 (1H, s, C-5’’H), 8.42 (1H, d, *J* = 15.26 Hz, C-1’H).

^{13}C NMR (100 MHz, $CDCl_3$): δ 38.95 (C-4a), 115.22, 120.47, 123.04, 125.15, 128.81, 129.65, 132.56, 139.08, 140.49, 142.87, 164.02 (C-2), 189.42 (C-3’).

HRMS: *m/z* Calculated for $C_{27}H_{18}FClN_4O_2$ $[M]^+$ 484.1123 found 484.1125.

(*E*)-1-((1-(4-chlorophenyl)-1*H*-1,2,3-triazol-4-yl)methyl)-3-(3-(4-fluorophenyl)-3-oxoprop-1-en-1-yl)quinolin-2(1*H*)-one (52)

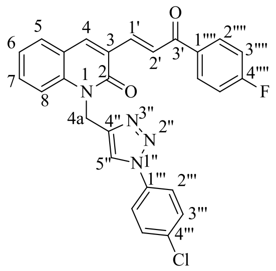

52

Yield: 75%, yellow solid.

Melting Point: 216-218 °C.

IR (KBr) (cm^{-1}): ν 3015 (sp^3 C-H), 1685 (C=O), 1620 (C=C), 1099 (C-F), 810 (*p*-substitution).

^{1}H NMR (400 MHz, $CDCl_3$): δ 5.70 (2H, s, C-4aH), 7.13-7.17 (2H, m, Ar-H), 7.22-7.27

(3H, m, Ar-H), 7.37-7.39 (1H, m, Ar-H), 7.43-7.51 (3H, m, Ar-H), 7.61-7.67 (3H, m, Ar-H), 7.79 (1H, d, *J* = 15.26 Hz, C-2'H), 7.81-7.83 (2H, m, Ar-H), 7.99 (1H, s, C-5''H), 8.10-8.01-8.02 (1H, m, Ar-H), 8.08-8.12 (3H, m, Ar-H), 8.45 (1H, d, *J* = 15.26 Hz, C-1'H).

^{13}C NMR (100 MHz, $CDCl_3$): δ 38.54 (C-4a), 115.13, 117.13, 125.56, 123.03, 129.71, 129.82, 130.91, 132.44, 139.51, 141.52, 160.92 (C-2), 189.64 (C-3').

HRMS: *m/z* Calculated for $C_{27}H_{18}FClN_4O_2$ $[M]^+$ 484.1123 found 484.1123.

(*E*)-1-((1-(4-chlorophenyl)-1*H*-1,2,3-triazol-4-yl)methyl)-3-(3-(4-chlorophenyl)-3-oxoprop-1-en-1-yl)quinolin-2(1*H*)-one (53)

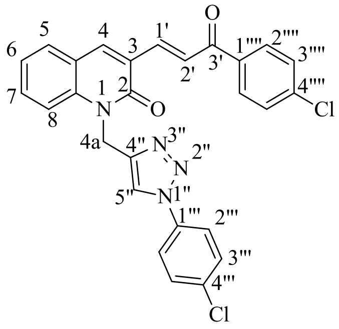

53

Yield: 95%, light green solid.

Melting Point: 180-182 °C.

IR (KBr) (cm^{-1}): ν 3025 (sp^3 C-H), 1685 (C=O), 1599 (C=C), 822 (*p*-substitution).

^{1}H NMR (400 MHz, $CDCl_3$): δ 5.70 (2H, s, C-4aH), 7.24-7.27 (2H, m, Ar-H), 7.44-7.46 (4H, m ,Ar-H), 7.63-7.65 (4H, m, Ar-H), 7.79 (1H, d, *J* = 12.97 Hz, C-2'H), 8.02-8.08 (4H, m, Ar-H), 8.42 (1H, d, *J* = 11.44 Hz, C-1').

^{13}C NMR (100 MHz, $CDCl_3$): δ 38.58 (C-4a), 115.18, 116.54, 116.77, 120.31, 121.99, 122.41, 122.49, 123.09, 125.18, 125.59, 128.88, 129.72, 130.07, 132.56, 136.29, 139.07, 139.50, 140.26, 142.82 51, 144.05, 160.88 (C-2), 163.21, 189.56 (C-3').

HRMS: *m/z* Calculated for $C_{27}H_{18}Cl_2N_4O_2$ $[M]^+$ 500.0856 found 500.0832.

(*E*)-3-(3-(4-bromophenyl)-3-oxoprop-1-en-1-yl)-1-((1-(4-chlorophenyl)-1*H*-1,2,3-triazol-4-yl)methyl)quinolin-2(1*H*)-one (**54**)

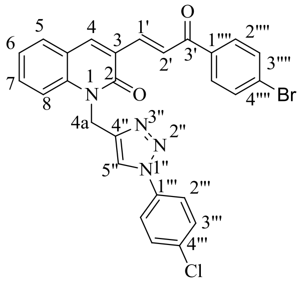

54

Yield: 85%, light yellowsolid.

Melting Point: 190-192 °C.

IR (KBr) (cm^{-1}): ν 2099 (sp^3 C-H), 1680 (C=O), 1609 (C=C), 822 (*p*-substitution).

^{1}H NMR (400 MHz, $CDCl_3$): δ 5.66 (2H, s, C-4aH), 7.24-7.29 (2H, m, Ar-H), 7.44-7.51 (2H, m ,Ar-H), 7.64-7.81 (6H, m, Ar-H), 7.95-8.13 (4H, m, Ar-H), 8.39-8.44 (1H, m, Ar-H),

^{13}C NMR (100 MHz, $CDCl_3$): δ 38.58 (C-4a), 115.18, 116.54, 116.77, 120.31, 121.99, 122.41, 122.49, 123.09, 125.18, 125.59, 128.88, 129.72, 130.07, 132.56, 136.29, 139.07, 139.50, 140.26, 142.82 51, 144.05, 160.88 (C-2), 163.21, 189.56 (C-3').

HRMS: *m/z* Calculated for $C_{27}H_{18}BrClN_4O_2$ $[M]^+$ 544.0302 found 544.0332.

(*E*)-1-((1-(4-chlorophenyl)-1*H*-1,2,3-triazol-4-yl)methyl)-3-(3-oxo-3-(*p*-tolyl)prop-1-en-1-yl)quinolin-2(1*H*)-one (**55**)

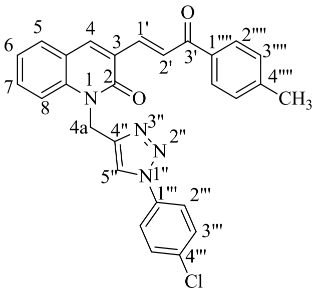

55

Yield: 65%, light green solid.

Melting Point: 217-219 °C.

IR (KBr) (cm^{-1}): ν 2956 (sp^3 C-H), 1695 (C=O), 1615 (C=C), 812 (*p*-substitution).

^{1}H NMR (400 MHz, $CDCl_3$): δ 2.40 (3H, s, -CH_3), 5.69 (2H, s, C-4aH), 7.25-7.27 (3H, m, Ar-H), 7.40-7.42 (3H, m, Ar-H), 7.57-7.61 (3H, m, Ar-H), 7.77 (1H, d, *J* = 15.11 Hz, C-2'H), 7.96-7.98 (3H, m, Ar-H), 8.13 (1H, s, C-5''H), 8.42 (1H, d, *J* = 15.11 Hz, C-1'H).

^{13}C NMR (100 MHz, $CDCl_3$): δ 21.67 (-$\underline{C}H_3$) 38.56 (C-4a), 121.59, 123.00, 125.76, 128.78, 129.95, 129.63, 129.82, 132.31, 134.54, 139.27, 142.18, 160.51 (C-2),

189.53 (C-3’).

HRMS: *m/z* Calculated for $C_{28}H_{21}ClN_4O_2$ $[M]^+$ 480.1446 found 480.1425.

(*E*)-1-((1-(4-chlorophenyl)-1*H*-1,2,3-triazol-4-yl)methyl)-3-(3-(2-methoxyphenyl)-3-oxoprop-1-en-1-yl)quinolin-2(1*H*)-one (**56**)

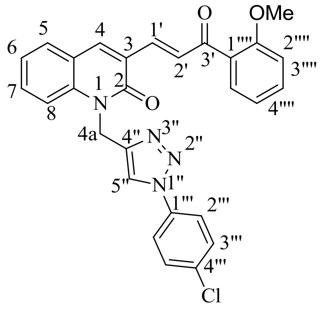

56

Yield: 75%, yellow solid.

Melting Point: 230-232 °C.

IR (KBr) (cm^{-1}): ν 2950 (sp^3 C-H), 1685 (C=O), 1610 (C=C), 815 (*p*-substitution).

^{1}H NMR (400 MHz, $CDCl_3$): δ 3.89 (3H, s, -OCH_3), 5.65 (2H, s, C-4aH), 6.96-7.02 (2H, m, Ar-H), 7.22-7.25 (1H, m, Ar-H), 7.40-7.46 (4H, m, Ar-H), 7.58-7.64 (5H, m, Ar-H), 7.69-7.73 (2H, m, Ar-H), 7.93-7.97 (2H, m, Ar-H), 8.08 (1H, s, C-5’’H).

^{13}C NMR (100 MHz, $CDCl_3$): δ 38.48 (C-4a), 55.78 (-O$\underline{C}H_3$), 111.62, 115.02, 120.62, 121.53, 122.93, 126.31, 129.56, 129.81, 130.25, 132.11, 132.87, 134.53, 139.05, 140.34, 157.67, 160.87 (C-2), 192.80 (C-3’).

HRMS: *m/z* Calculated for $C_{28}H_{21}ClN_4O_3$ $[M]^+$ 496.1446 found 496.1425.

(*E*)-1-((1-(4-chlorophenyl)-1*H*-1,2,3-triazol-4-yl)methyl)-3-(3-(3-methoxyphenyl)-3-oxoprop-1-en-1-yl)quinolin-2(1*H*)-one (**57**)

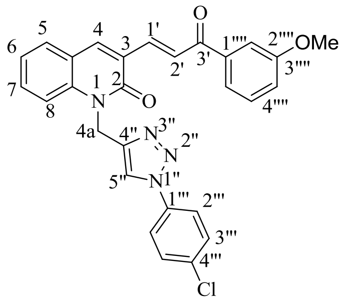

57

Yield: 65%, yellow solid.

Melting Point: 215-217 °C.

IR (KBr) (cm^{-1}): ν 2852 (sp^3 C-H), 1690 (C=O), 1630 (C=C), 822 (*p*-substitution).

^{1}H NMR (400 MHz, $CDCl_3$): δ 3.85 (3H, s, -OCH_3), 5.68 (2H, s, C-4aH), 7.09-7.11 (1H, m, Ar-H), 7.24-7.26 (1H, m, Ar-H), 7.39-7.45 (3H, m, Ar-H), 7.57-7.67 (5H, m, Ar-H), 7.78 (1H, d, *J* = 15.57 Hz, C-2’), 7.94-7.98 (1H, m, Ar-H), 8.13 (1H, s, C-5’’H), 8.38 (1H, d, *J* = 15.11 Hz, C-1’).

^{13}C NMR (100 MHz, $CDCl_3$): δ 38.53 (C-4a), 55.44 (-OC̲H₃), 112.79, 115.10, 119.41, 120.31, 121.36, 121.57, 123.02, 125.72, 129.51, 129.83, 132.38, 139.01, 139.34, 139.71, 142.21, 159.79, 160.87 (C-2), 191.31 (C-3').

HRMS: *m/z* Calculated for $C_{28}H_{21}ClN_4O_3$ $[M]^+$ 496.1302 found 496.1300.

(*E*)-1-((1-(4-chlorophenyl)-1*H*-1,2,3-triazol-4-yl)methyl)-3-(3-(4-methoxyphenyl)-3-oxoprop-1-en-1-yl)quinolin-2(1*H*)-one (**58**)

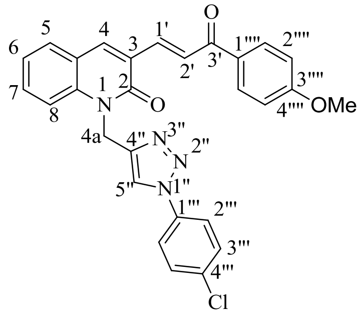

58

Yield: 85%, yellow solid.

Melting Point: 210-212 °C.

IR (KBr) (cm^{-1}): ν 2950 (sp^3 C-H), 1685 (C=O), 1599 (C=C), 820(*p*-substitution).

^{1}H NMR (400 MHz, $CDCl_3$): δ 3.85 (3H, s, $-OCH_3$), 5.68 (2H, s, C-4aH), 7.09-7.11 (1H, m, Ar-H), 7.24-7.26 (1H, m, Ar-H), 7.39-7.45 (3H, m, Ar-H), 7.57-7.67 (5H, m, Ar-H), 7.78 (1H, d, *J* = 15.57 Hz, C-2'), 7.94-7.98 (1H, m, Ar-H), 8.13 (1H, s, C-5''H), 8.38 (1H, d, *J* = 15.11 Hz, C-1').

^{13}C NMR (100 MHz, $CDCl_3$): δ 38.07 (C-4a), 55.48 (-OC̲H₃), 113.78, 115.09, 115.51, 119.43, 121.56, 121.73, 121.89, 123.49, 124.93, 129.62, 129.84, 134.36, 134.68, 138.97, 142.26, 143.82, 161.67 (C-2), 189.66 (C-3').

HRMS: *m/z* Calculated for $C_{28}H_{21}ClN_4O_3$ $[M]^+$ 496.1455 found 496.1425.

(*E*)-3-(3-(2-fluorophenyl)-3-oxoprop-1-en-1-yl)-1-((1-phenyl-1*H*-1,2,3-triazol-4-yl)methyl)quinolin-2(1*H*)-one (**59**)

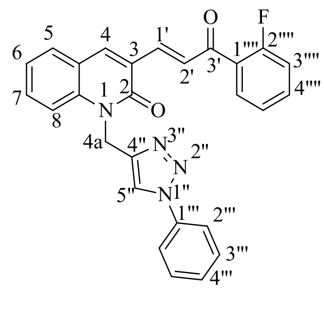

59

Yield: 70%, yellow solid.

Melting Point: 172-174 °C.

IR (KBr) (cm^{-1}): ν 2995 (sp^3 C-H), 1695 (C=O), 1590 (C=C), 1090 (C-F).

^{1}H NMR (400 MHz, $CDCl_3$): δ 5.69 (2H, s, C-4aH), 7.13-7.17 (2H, m, Ar-H), 7.22-

7.27 (3H, m, Ar-H), 7.37-7.39 (1H, m, Ar-H), 7.43-7.51 (3H, m, Ar-H), 7.61-7.67 (3H, m, Ar-H), 7.79-7.83 (2H, m, Ar-H), 7.99 (1H, s, C-5''H), 8.01-8.02 (1H, m, Ar-H), 8.11-8.12 (2H, m, Ar-H).

^{13}C NMR (100 MHz, $CDCl_3$): δ 38.54 (C-4a), 115.13, 117.13, 125.56, 123.03, 129.71, 129.82, 130.91, 132.44, 139.51, 141.52, 160.92 (C-2), 189.64 (C-3').

HRMS: *m/z* Calculated for $C_{27}H_{19}FN_4O_2$ $[M]^+$ 450.1587 found 450.1568.

(*E*)-3-(3-(3-fluorophenyl)-3-oxoprop-1-en-1-yl)-1-((1-phenyl-1*H*-1,2,3-triazol-4-yl)methyl)quinolin-2(1*H*)-one (60)

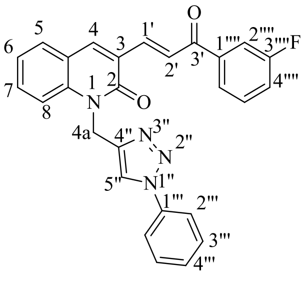

60

Yield: 90%, yellow solid.

Melting Point: 180-182 °C.

IR (KBr) (cm^{-1}): ν 2936 (sp^3 C-H), 1660 (C=O), 1599 (C=C) 1028 (C-F).

^{1}H NMR (400 MHz, $CDCl_3$): δ 5.70 (2H, s, C-4aH), 7.25-7.27 (3H, m, Ar-H), 7.38-7.44 (4H, m, Ar-H), 7.63-7.67 (3H, m, Ar-H), 7.73-7.78 (3H, m, Ar-H), 7.85-7.87 (2H, m, Ar-H), 8.00 (1H, s, C-5''H), 8.40 (1H, d, *J* = 15.11 Hz, C-1'H).

^{13}C NMR (100 MHz, $CDCl_3$): δ 37.99 (C-4a), 115.22, 120.47, 123.04, 125.15, 128.81, 129.65, 132.56, 139.08, 140.49, 142.87, 161.01, 164.02 (C-2), 189.42 (C-3').

HRMS: *m/z* Calculated for $C_{27}H_{19}FN_4O_2$ $[M]^+$ 450.1587 found 450.1500.

(*E*)-3-(3-(4-fluorophenyl)-3-oxoprop-1-en-1-yl)-1-((1-phenyl-1*H*-1,2,3-triazol-4-yl)methyl)quinolin-2(1*H*)-one (61)

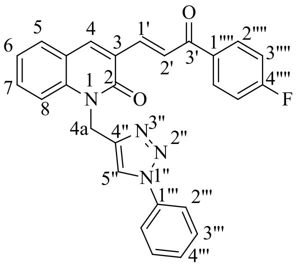

61

Yield: 80%, light yellow solid.

Melting Point: 202-204 °C.

IR (KBr) (cm^{-1}): ν 2956 (sp^3 C-H), 2855, 1665 (C=O), 1599 (C=C), 1033 (C-F), 820 (*p*-substitution).

^{1}H NMR (400 MHz, $CDCl_3$): δ 5.71 (2H, s, C-4aH), 7.16-7.20 (2H, m, Ar-H), 7.25-7.27 (1H, m, Ar-H), 7.39-7.40 (1H, m, Ar-H), 7.45-7.47 (2H, m, Ar-H), 7.66-7.70 (4H, m, Ar-H), 7.78 (1H, d, *J* = 15.57 Hz, C-2’H), 7.99-8.00 (1H, m, Ar-H), 8.11-8.14 (3H, m, Ar-H), 8.46 (1H, d, *J* = 15.11 Hz, C-1’H).

^{13}C NMR (100 MHz, $CDCl_3$): δ 38.56 (C-4a), 115.18, 116.54, 116.77, 120.31, 121.99, 122.41, 122.49, 123.09, 125.18, 125.59, 128.88, 129.72, 130.07, 132.56, 136.29, 139.07, 139.50, 140.26, 142.82 51, 144.05, 160.88 (C-2), 189.19 (C-3’).

HRMS: *m/z* Calculated for $C_{27}H_{19}BrN_4O_2$ $[M]^+$ 510.0756 found 510.0717.

(*E*)-3-(3-(4-chlorophenyl)-3-oxoprop-1-en-1-yl)-1-((1-phenyl-1*H*-1,2,3-triazol-4-yl)methyl)quinolin-2(1*H*)-one (62)

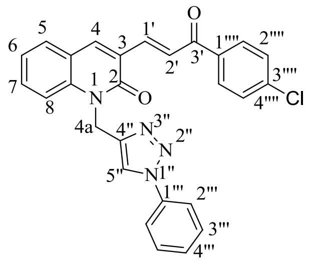

62

Yield: 75%, light yellow solid.

Melting Point: 216-218 °C.

IR (KBr) (cm^{-1}): ν 2925 (sp^3 C-H), 1710 (C=O), 1600 (C=C), 822 (*p*-substitution).

^{1}H NMR (400 MHz, $CDCl_3$): δ 5.72 (2H, s, C-4aH), 7.28-7.30 (1H, m, Ar-H), 7.37-7.40 (2H, m, Ar-H), 7.42-7.69 (2H, m, Ar-H), 7.62-7.68 (4H, m, Ar-H), 7.80 (1H, d, *J* = 13.73 Hz, C-2’H), 7.95-7.97 (3H, m, Ar-H), 8.02 (1H, s, C-4H), 8.11 (1H, s, C-5’’H), 8.44 (1H, d, *J* = 13.73 Hz, C-1’).

^{13}C NMR (100 MHz, $CDCl_3$): δ 38.58 (C-4a), 115.18, 116.54, 116.77, 120.31, 121.99, 122.41, 122.49, 123.09, 125.18, 125.59, 128.88, 129.72, 130.07, 132.56, 136.29, 139.07, 139.50, 140.26, 142.82 51, 144.05, 162.32 (C-2), 189.56 (C-3’).

HRMS: *m/z* Calculated for $C_{27}H_{19}ClN_4O_2$ $[M]^+$ 466.1197 found 466.1127.

(*E*)-3-(3-(4-bromophenyl)-3-oxoprop-1-en-1-yl)-1-((1-phenyl-1*H*-1,2,3-triazol-4-yl)methyl)quinolin-2(1*H*)-one (63)

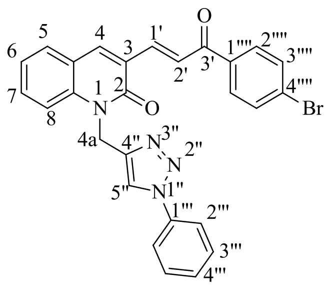

63

Yield: 80%, light yellow solid.

Melting Point: 212-214 °C.

IR (KBr) (cm^{-1}): ν 3025 (sp^3 C-H), 1715 (C=O), 1619 (C=C), 832 (*p*-substitution).

^{1}H NMR (400 MHz, $CDCl_3$): δ 5.72 (2H, s, C-4aH), 7.28-7.30 (1H, m, Ar-H), 7.37-7.40 (2H, m, Ar-H), 7.42-7.69 (2H, m, Ar-H), 7.62-7.68 (4H, m, Ar-H), 7.80 (1H, d, *J* = 13.73 Hz, C-2'H), 7.95-7.97 (3H, m, Ar-H), 8.02 (1H, s, C-4H), 8.11 (1H, s, C-5''H), 8.44 (1H, d, *J* = 13.73 Hz, C-1').

^{13}C NMR (100 MHz, $CDCl_3$): δ 38.58 (C-4a), 115.18, 116.54, 116.77, 120.31, 121.99, 122.41, 122.49, 123.09, 125.18, 125.59, 128.88, 129.72, 130.07, 132.56, 136.29, 139.07, 139.50, 140.26, 142.82 51, 144.05, 162.32 (C-2), 189.56 (C-3').

HRMS: *m/z* Calculated for $C_{27}H_{19}FN_4O_2$ $[M]^+$ 450.1587 found 450.1527.

(*E*)-3-(3-oxo-3-(*p*-tolyl)prop-1-en-1-yl)-1-((1-phenyl-1*H*-1,2,3-triazol-4-yl)methyl)quinolin-2(1*H*)-one (64)

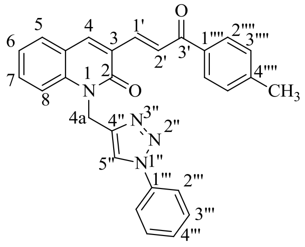

64

Yield: 90%, yellow solid.

Melting Point: 216-218 °C.

IR (KBr) (cm^{-1}): ν 2936 (sp^3 C-H), 1685 (C=O), 1590 (C=C), 818 (*p*-substitution).

^{1}H NMR (400 MHz, $CDCl_3$): δ 2.40 (3H, s, $-CH_3$), 5.72 (2H, s, C-4aH), 7.25-7.29 (3H, m, Ar-H), 7.30-7.47 (4H, m, Ar-H), 7.61-7.67 (4H, m, Ar-H), 7.69 (1H, d, *J* = 15.11 Hz, C-2'H), 7.99-8.01 (3H, m, Ar-H), 8.14 (1H, s, C-5''H), 8.46 (1H, d, *J* = 15.11 Hz, C-1'H).

^{13}C NMR (100 MHz, $CDCl_3$): δ 21.67 ($-\underline{C}H_3$) 38.57 (C-4a), 121.59, 123.00, 125.76, 128.78, 129.95, 129.63, 129.82, 132.31, 134.54, 139.27, 142.18, 169.22 (C-2),

189.84 (C-3').

HRMS: *m/z* Calculated for $C_{28}H_{22}N_4O_2$ $[M]^+$ 446.1745 found 446.1745.

(*E*)-3-(3-(2-methoxyphenyl)-3-oxoprop-1-en-1-yl)-1-((1-phenyl-1*H*-1,2,3-triazol-4-yl)methyl)quinolin-2(1*H*)-one (65)

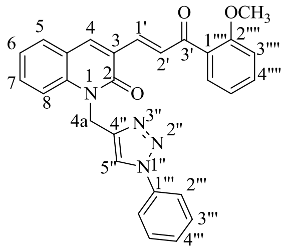

65

Yield: 90%, light yellow solid.

Melting Point: 210-212 °C.

IR (KBr) (cm^{-1}): ν 2990 (sp^3 C-H), 1680 (C=O), 1589 (C=C), 1099 (C-O-C).

^{1}H NMR (400 MHz, $CDCl_3$): δ 3.89 (3H, s, -OCH_3), 5.67 (2H, s, C-4aH), 6.98-7.24 (2H, m, Ar-H), 7.25-7.27 (2H, m, Ar-H), 7.42-7.46 (4H, m, Ar-H), 7.58-7.61 (3H, m, Ar-H), 7.64-7.66 (2H, m, Ar-H), 7.72 (1H, d, *J* = 15.57, C-2'H), 7.94-7.98 (3H, m, Ar-H), 8.08 (1H, s, C-5''H).

^{13}C NMR (100 MHz, $CDCl_3$): δ 38.57 (C-4a), 55.78 (-O$\underline{C}H_3$), 111.63, 115.11, 120.40, 120.62, 122.88, 126.34, 128.77, 129.53, 129.63, 130.25, 132.10, 132.84, 138.05, 139.11, 140.36, 158.14, 161.10, 193.06 (C-3').

HRMS: *m/z* Calculated for $C_{28}H_{22}N_4O_3$ $[M]^+$ 462.1732 found 462.1746.

(*E*)-3-(3-(3-methoxyphenyl)-3-oxoprop-1-en-1-yl)-1-((1-phenyl-1*H*-1,2,3-triazol-4-yl)methyl)quinolin-2(1*H*)-one (66)

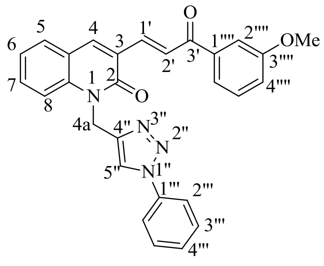

66

Yield: 92%, yellow solid.

Melting Point: 224-226 °C.

IR (KBr) (cm^{-1}): ν 3025 (sp^3 C-H), 1680 (C=O), 1592 (C=C), 1100 (C-O-C).

^{1}H NMR (400 MHz, $CDCl_3$): δ 3.87 (3H, s, -OCH_3), 5.71 (2H, s, C-4aH), 7.10-7.13 (1H, m, Ar-H), 7.24-7.28 (1H, m, Ar-H), 7.38-7.47 (5H, m, Ar-H), 7.59-7.70 (5H, m, Ar-H), 7.81 (1H, d, *J* = 15.26 Hz, C-2'H), 7.98-8.01 (2H, m, Ar-H), 8.11 (1H, s, C-

5''H), 8.41 (1H, d, *J* = 15.26 Hz, C-1'H).

^{13}C NMR (100 MHz, $CDCl_3$): δ 38.53 (C-4a), 55.44 (-O<u>C</u>H_3), 112.79, 115.10, 119.41, 120.31, 121.36, 121.57, 123.02, 125.72, 125.80, 129.83, 132.38, 139.01, 139.34, 139.71, 142.21, 159.79, 160.87 (C-2), 190.77 (C-3').

HRMS: *m/z* Calculated for $C_{28}H_{22}N_4O_3$ $[M]^+$ 462.1732 found 462.1743.

(*E*)-3-(3-(4-methoxyphenyl)-3-oxoprop-1-en-1-yl)-1-((1-phenyl-1*H*-1,2,3-triazol-4-yl)methyl)quinolin-2(1*H*)-one (67)

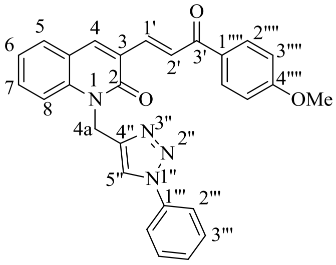

67

Yield: 93%, light yellow solid.

Melting Point: 215-217 °C.

IR (KBr) (cm^{-1}): ν 2935 (sp^3 C-H), 1667 (C=O), 1605 (C=C), 1090 (C-O-C), 820 (*p*-substitution).

^{1}H NMR (400 MHz, $CDCl_3$): δ 3.87 (3H, s, -OCH_3), 5.71 (2H, s, C-4aH), 6.95 (2H, d, *J* = 7.25 Hz, Ar-H), 7.38-7.45 (4H, m, Ar-H), 7.63-7.66 (3H, m, Ar-H), 7.78 (1H, d, *J* = 15.26 Hz, C-1'H), 7.97-7.99 (2H, m, Ar-H), 8.11-8.11 (2H, m, Ar-H), 8.47 (1H, d, *J* = 15.26 Hz, C-1'H).

^{13}C NMR (100 MHz, $CDCl_3$): δ 38.07 (C-4a), 55.48 (-O<u>C</u>H_3), 113.78, 115.09, 115.51, 119.43, 121.56, 121.73, 121.89, 123.49, 124.93, 129.62, 129.84, 129.88, 131.04, 131.88, 132.29, 134.36, 134.68, 138.97, 141.27, 142.26, 143.82, 161.31 (C-2), 189.66 (C-3').

HRMS: *m/z* Calculated for $C_{28}H_{22}N_4O_3$ $[M]^+$ 462.1732 found 462.1721.
